# *Helicobacter pylori* HP0377, a member of the Dsb family, is an untypical multifunctional CcmG that cooperates with dimeric thioldisulfide oxidase HP0231

**DOI:** 10.1186/s12866-015-0471-z

**Published:** 2015-07-04

**Authors:** Paula Roszczenko, Magdalena Grzeszczuk, Patrycja Kobierecka, Ewa Wywial, Paweł Urbanowicz, Piotr Wincek, Elzbieta Nowak, E. Katarzyna Jagusztyn-Krynicka

**Affiliations:** Department of Bacterial Genetics, Institute of Microbiology, Faculty of Biology, University of Warsaw, Warsaw, Poland; Laboratory of Bioinformatics and Protein Engineering, International Institute of Molecular and Cell Biology, Warsaw, Poland; Laboratory of Protein Structure, International Institute of Molecular and Cell Biology, Warsaw, Poland; Present address: Department of Cell Biology, Nencki Institute of Experimental Biology, PAS, Warsaw, Poland

**Keywords:** Dsb, *Helicobacter pylori*, Cytochrome c biogenesis, Isomerase activity

## Abstract

**Background:**

In the genome of *H. pylori* 26695, 149 proteins containing the CXXC motif characteristic of thioldisulfide oxidoreductases have been identified to date. However, only two of these proteins have a thioredoxin-like fold (i.e., HP0377 and HP0231) and are periplasm-located. We have previously shown that HP0231 is a dimeric oxidoreductase that catalyzes disulfide bond formation in the periplasm. Although HP0377 was originally described as DsbC homologue, its resolved structure and location of the *hp0377* gene in the genome indicate that it is a counterpart of CcmG/DsbE.

**Results:**

The present work shows that HP0377 is present in *H. pylori* cells only in a reduced form and that absence of the main periplasmic oxidase HP0231 influences its redox state. Our biochemical analysis indicates that HP0377 is a specific reductase, as it does not reduce insulin. However, it possesses disulfide isomerase activity, as it catalyzes the refolding of scrambled RNase. Additionally, although its standard redox potential is -176 mV, it is the first described CcmG protein having an acidic pKa of the N-terminal cysteine of the CXXC motif, similar to *E. coli* DsbA or *E. coli* DsbC. The CcmG proteins that play a role in a cytochrome c-maturation, both in system I and system II, are kept in the reduced form by an integral membrane protein DsbD or its analogue, CcdA. In *H. pylori* HP0377 is re-reduced by CcdA (HP0265); however in *E. coli* it remains in the oxidized state as it does not interact with *E. coli* DsbD. Our *in vivo* work also suggests that both HP0377, which plays a role in apocytochrome reduction, and HP0378, which is involved in heme transport and its ligation into apocytochrome, provide essential functions in *H. pylori*.

**Conclusions:**

The present data, in combination with the resolved three-dimensional structure of the HP0377, suggest that HP0377 is an unusual, multifunctional CcmG protein.

**Electronic supplementary material:**

The online version of this article (doi:10.1186/s12866-015-0471-z) contains supplementary material, which is available to authorized users.

## Background

Disulfide bonds contribute to the stability and function of many extracytoplasmic, soluble or membrane-bound proteins. In gram-negative bacteria, the oxidative protein folding takes place in the periplasm and is controlled by proteins from the Dsb family [1]. In the highly oxidizing environment of the periplasm, there is also a need for selected proteins to be kept in a reduced form. In the assembly of c-type cytochromes, essential for energy metabolism, for example, the cytochrome c maturation process requires ligation of heme (iron protoporphirin IX) to reduced thiols of the Cys-X-X-Cys-His motif of apocytochrome [2]. To date, there are two different systems for cytochrome-c biogenesis that have been found in bacteria: system I and system II. They comprise two kinds of proteins acting in a coordinated fashion: those involved in heme handling and heme ligation to the apocytochrome, and those contributing to reduction of a disulfide bond of the CXXCH heme-binding motifs [3–5].

The cytochrome-c maturation machinery of the system I operating in many gram-negative bacteria consists of up to ten proteins (Ccm ABCDEFGHI and DsbD or its shorter version CcdA). The apocytochrome reduction is accomplished by the action of CcmG (also known as DsbE), CcmH, and DsbD or CcdA. DsbD/CcdA transfers electrons from cytoplasmic thioredoxin to CcmG. Then CcmG is re-oxidized by shuttling its electrons to CcmH, which finally transfers them to apocytochrome c. However, the details concerning mixed disulfide complexes created during the process still remain unclear [6]. The remaining proteins play a role in heme transport and its ligation to the apocytochrome [4].

System II is simpler and more widespread among bacterial species. It is present in, for example, gram-positive *Bacillus subtilis.* It contains four proteins, ResA, ResB, ResC and CcdA, of which ResA (also named CcsX or HelX) is a functional counterpart of CcmG, and CcdA is a functional counterpart of DsbD. ResB (also called CcsB and Ccs1) and ResC (CcsA) form a complex to deliver the heme, and they function in the cytochrome c-heme ligation [7]. While in most microorganisms, the CcsB and CcsA proteins are present as two separate polypeptides, there are a few species of bacteria, such as *Wolinella, Bacteroides* and some strains *of Helicobacter spp.,* whose genes encode CcsA and CcsB fused into one large ORF (called *ccsBA*) [8].

Many studies have tried to decipher the cooperation among periplasmic Dsb proteins in cytochrome-c biogenesis. Initially, it was thought that introduction of the disulfide bonds into the CXXCH motif of the apocytochrome c, which happens just after its transport to the periplasm by the Sec system, is an obligatory step of the process, as *dsbA* and *dsbB* mutants in *E. coli* were unable to produce cytochrome c [9, 10]. However, recent data in the literature may contradict this scheme; the lack of the Dsb proteins of the oxidative pathway in *B. subtilis* and *R. capsulatus,* for example, suppresses the cytochrome c deficiency of *ccmG* or *ccdA* mutants [11–13]. However, it should be noted that the Dsb oxidative pathway functioning is dependent on cell growth conditions (anaerobic vs aerobic) It has also been demonstrated that the heterologous expression of CcsBA of *H. pylori* or CcsAB from *B. pertussis,* both encoding cytochrome c synthetase*,* in an *E. coli* that lacks its own cytochrome c machinery results in c-type cytochrome formation. This observed effect was significantly enhanced by addition of exogenous reductant [14, 15]. Detailed analysis of the *Paracoccus denitrificans* cytochrome maturation in an *E. coli dsbA, dsbD* double-mutant led Mavridou et al. to the conclusion that apocytochrome is subjected to two competing reactions: either heme attachment to its cysteine thiols, or oxidation of those thiols [16].

*Helicobacter pylori* is a gram-negative spiral-shaped bacterium, a member of ε-Proteobacteria that specifically colonizes the gastric epithelium of humans with severe consequences. Data on *H. pylori* cytochromes are not comprehensive. Analysis of its genome nucleotide sequence revealed that this microaerophilic microorganism possesses a rather simple respiratory chain consisting of three enzymes: quinol-cytochrome c reductase, cytochrome bc_1_ complex, cytochrome c_553_ and cb-type cytochrome c oxidase [17]. Further inspection of the *H. pylori* genome showed the existence of a second gene encoding a putative low molecular mass cytochrome c (HP0236) named *cycB*. Both *cycA* and *cycB* are essential genes and cannot substitute for each other [18]. Cytochrome c_553_, encoded by the *cycA* (HP1227) gene, is a soluble periplasmic protein, a potential electron donor to the *cb*-type cytochrome *c* oxidase [18]. To our knowledge, little is known about the c-type cytochrome maturation system of *Helicobacter pylori*. In the genome of *H. pylori* 26695, there are 149 proteins containing CXXC motifs characteristic of thiol:disulfide oxidoreductases identified to date. Only two of these proteins have a thioredoxin-like (TRX) fold (i.e., HP0377 and HP0231) and are periplasm-located [19]. This microorganism uses system II, and *hp0377* (a homolog of *resA*) and *hp0378* ((*ycf5*), a homolog of *resBC*), whose products potentially play a central role in c-type cytochrome maturation, constitute one transcriptional unit [20, 21]. HP0378 can, to some extent, complement for a lack of the *E. coli* cytochrome-c maturation machinery. The recently determined crystal structure of HP0377 shows similarity to the structure of *B. subtilis* ResA. However large structural differences between these two proteins were also observed [22]. We have previously demonstrated that HP0231 is a dimeric oxidoreductase that catalyzes disulfide bond formation in the periplasm. In this work, to gain further insight into the *Helicobacter pylori* Dsb protein network, we analyzed HP0377, the second periplasmic oxidoreductase, using biochemical and genetic tools.

## Methods

### Bacterial strains, primers, plasmids, media and growth conditions

Bacterial strains, plasmids and primers used in this study are listed in Tables 1 and 2. Two *H. pylori* strains (26695 and N6) were used in this study. Although the sequence of the *H. pylori* 26695 genome is complete [23], this strain is inconvenient for genetic manipulation. *H. pylori* N6, originally isolated from a patient with gastritis, is useful for complementation experiments, and it is highly motile [24]. Both *H. pylori* strains were grown on blood agar base 2 (BA) plates (Merck) supplemented with 10 % horse blood and an antibiotic mixture consisting of vancomycin (final concentration, 12.5 μg ml^−1^), polymyxin B (1.25 μg ml^−1^), trimethoprim (6.25 μg ml^−1^) and amphotericin B (2.5 μg ml^−1^) at 37 °C under microaerobic conditions. Liquid cultures of *H. pylori* were grown in Brain Heart Infusion (BHI) broth supplemented with 10 % fetal bovine serum (FBS). For the selection of *H. pylori* mutants or complemented strains, kanamycin (25 μg ml^−1^) or/and chloramphenicol (10 μg ml^−1^) were added to the growth media.

**Table 1:** Bacterial strains and plasmids used in this study

Name	Genotype or relevant characteristics	Origin
*Helicobacter pylori* strains		
26696	*H. pylori* wild-type	ATCC
N6	*H. pylori* wild-type	[24]
PR305	N6 dsbI::*aph*	[31]
PR336	*dsbI* ^*+*^ in trans complementant of *dsbI::aph*	[31]
PR378	N6 *hp0231::cat*	[31]
PR397	*hp0231* ^*+*^ in trans complementant of *hp0231::cat*	[31]
MG100	N6 *hp0265::aph*	This study
*Escherichia coli* strains		
BL21 (DE3)	F*- ompT hsdSB*(*rB-mB-*) *gal dcm lon*	Novagen
Rosetta (DE3) LacIq	F- *ompT hsdSB (rB- mB-) gal dcm* pRARE (Cm^r^)	Novagen
TG1	*supE44 hsd*Δ *5 thi* Δ(*lac- proAB*) F’ [*traD36 proAB + lacIq lacZ*ΔM15]	[26]
Top10	F- *mcrA* Δ(*mrr-hsdRMS-mcrBC*) φ80*lacZ* ΔM15 Δ*lacX74 deoR nupG recA1 araD139* Δ(*ara-leu*)7697 *galU galK rpsL*(str^r^) *endA1* λ-	Gibco BRL
JCB816	MC1000 *phoR* λ102	[72]
JCB817	JCB816 *dsbA*::*kan1*	[72]
JFC383	JCB816 *dsbC*::*kan*	J.F. Collet Collection
FED126	MC1000 Δ*dsbD*	[73]
JFC571	JFC383/pHEL2	This study
JFC572	JCF383/pUWM500	This study
JFC573	JF383/pUWM399 *hp0377* ^*+*^	This study
PR501	JCB817/pHEL2	This study
PR502	JCB817/pUWM399 *hp0377* ^*+*^	This study
PR539	FED126/pUWM399 *hp0377* ^*+*^ & pUWM536	This study
PR540	JCB816/pUWM399 *hp0377* ^*+*^ & pUWM536	This study
PR580	FED126/pUWM399 *hp0377* ^*+*^	This study
PR581	JCB816/pUWM399 *hp0377* ^*+*^	This study
General cloning/Plasmid vectors		
pET28a	Km^r^, IPTG inducible	Novagen
pGEM-T Easy	Ap^r^; LacZa	Promega
pBlusescript SK II	Amr, LacZa	Stratagene
pHEL2	Cm^r^ *E. coli/H. pylori* shuttle vector	[74]
pMPM-K6Ω	Km^r^,Sp^r^; *ori* f1, *ori* P15A; arabinose inducible	[75]
pRY109	Cm^r^	[76]
pUOA13	Km^R^,Tet^R^	[77]
pILL2150	Cm^r^ *E.coli/H. pylori* shuttle vector; IPTG inducible	[47]
Plasmids for mutagenesis		
pUWM505	pGEM-T Easy/*hp0377::aph*	This study
pUWM529	pGEM-T Easy/*hp0377::aph*	This study
pUWM555	pGEM-T Easy/*hp0377::aph*	This study
pUWM2019	pBluescript II SK/*hp0265::aph*	This study
Plasmids for recombinant protein synthesis and purification		
pUWM518	pET28a/*hp0377*	This study
pUWM2046	pET28a/*hp0377* C89S	This study
pUWM2065	pET28a/*hp0377* C92S	This study
pUWM2090	pET24a/*hp1227*	This study
pET28a-dsbA	pET28a/*EcdsbA*	J.F. Collet Collection
pET28a-dsbC	pET28a/*EcdsbC*	J.F. Collet Collection
pUWM525	pET28a/*hp0231*	[31]
Other plasmids		
pUWM500	pHEL2/*hp0231*	[31]
pUWM336	pHEL2/*dsbI*	[78]
pUWM399	pHEL2/*hp0377*	This study
pUWM389	pGEM-T Easy/*hp0231*	This study
pUWM536	pMPM-K6Ω/*hp0265*	This study
pUWM543	pGEM-T easy/*hp0377*	This study
pUWM544	pGEM-T easy/hp0377 without promoter and signal sequence	This study
pUWM2045	pGEM-T easy/*hp0377* C89S	This study
pUWM2064	pBluescript II SK/*hp0377* C92S	This study
pUWM2073	pBluescript II SK/*hp0377* C25A	This study

**Table 2:** Primers used in this study

Name	Sequence 5′ – 3′	Orientation/Restriction site
hp377I	GGCGATACTTACCAGCAAG	Fwd/Ø
hp377II	ATCCACTTTTCAATCTATATCTGTCTATATTGTCTCGCTCATC	Rev/Ø
hp377IIa	CAAGGCAAT CTGCCTCCTCATGTCTATATTGTCTCGCTCATC	Rev/Ø
hp377IIIa	GGATGAATTGTTTTAGTACCAATTTATCGGCGATGGGAAG	Fwd/Ø
hp377IV	GTTTGGTGGTGTCATTAAGAGTG	Rev/Ø
hp377ex1	GAGGCCATGGGCAAATCCAACAATAAAGAC	Fwd/NcoI
hp377ex2	GTGCTCGAGGTTAGACTTGCTTTTAGAAAG	Rev/XhoI
HP0377zew1	CTAAGGTGCGAGTAATAGAG	Fwd/Ø
HP0377zew2	CTATGCCTTCATTCCTGTTG	Rev/Ø
hp0377mutF	GAGGCATATGATGTTTTCACTTTCTTATGTTTCC	Fwd/NdeI
hp0377mutR	GAGGATCCCTAGTTAGACTTGCTTTTAGAAAG	Rev/BamHI
N6_377_C89S_for	CTTTCGCAATAGGAGCTACCATTACGCCCAAAAACTAAAA	Fwd/Ø
N6_377_C89S_rev	CTTTCGCAATAGGAGCTACCATTACGCCCAAAAACTAAAA	Rev/Ø
hp377_C92SII	GTTTTTGGCCGTAATGGTTGCTCCTATTCCGAAAG	Fwd/Ø
hp377_C92SII_rev	CTTTCGGAATAGGAGCAACCATTACGGCCAAAAAC	Rev/Ø
hp377_C25A_for	ATTGATTTCGCTGTTTTTAAGCGCTGCCAAATCCAACAAT AAAGACAAGTTA	Fwd/Ø
hp377_C25A_rev	TAACTTGTCTTTATTGTTGGATTTGGCAGCGCTTAAAAAC AGCGAAATCAAT	Rev/Ø
N6_HP0264_fwd1	GATAGTAAGGGCGTGAGG	Fwd/Ø
N6_HP0265_up_Bam	AAGGGATCCGCGATTAAAGAGAGCTT	Fwd/BamHI
N6_HP0265_dw_Sal	TTAAGCGTAGCGATGTCGACTAGTTG	Rev/SalI
N6_HP0265_rev1	GGCGTAATGGCTGATGAG	Rev/Ø
N6_HP0265_up_rev	TAGCTGCAGCCCGGGTAGCCACCACAAACATCAAGGG	Rev/Ø
N6_HP0265_dw_fwd	CTACCCGGGCTGCAGCTATCCTTATGGTGGTGTTTG	Fwd/Ø
KM1f	TGAGGAGGCAGATTGCCTTG	Fwd/Ø
KM2r	GGTACTAAAACAATTCATCC	Rev/Ø
1hp265F-6HisNew	GAGCCATGGCACACCACCACCACCACCACATGTTTGATAACACGCTTG	Fwd/NcoI
2hp265RPstI	GTGCTGCAGCTATTTTTGCAAGAAATTCGTCAGAC	Rev/PstI
Hp1227_NdeI-for	GCGCATATGACCGATGTTAAAGCCCTTG	Fwd/NdeI
Hp1227_XhoI-rev	CGCTCGAGTTTGAGGGTGGGGATGTAT	Rev/XhoI

The *E. coli* strain TG1 was used as a host for the construction and preparation of recombinant plasmids. The *E. coli* strain Rosetta (DE3) was used to overexpress pUWM518, pUWM2046 and pUWM2065. The *E. coli* strains JCB817 and JFC383 were employed for complementation experiments of *E. coli dsbA* and *dsbC* mutants by HP0377. *E. coli* strains were grown at 37 °C on solid or liquid Luria-Bertani (LB) medium or on M63 minimal medium [25]. When needed, media were supplemented with antibiotics at the following concentrations: 100 μg ml^−1^ ampicillin, 30 μg ml^−1^ kanamycin and 20 μg ml^−1^ chloramphenicol.

### General DNA manipulations

Standard DNA manipulations were carried out as described earlier [26] or according to the manufacturer’s instructions (A&A Biotechnology). Polymerase chain reactions (PCR) were performed with PrimeStar HS DNA Polymerase (Takara) or HotStar HiFidelity Polymerase (Qiagen) under standard conditions. Synthetic oligonucleotides synthesis and DNA sequencing were performed by Genomed S.A., Warsaw, Poland.

### Natural transformation of *H. pylori*

The naturally competent *H. pylori* N6 was grown on BA plates for 24 h. Subsequently, bacteria were plated onto fresh plates for 5 h. Then 0.5–1 μg of plasmid DNA was added and plates were incubated for 22 h. Afterwards, bacteria were transferred onto a plate supplemented with kanamycin or chloramphenicol/kanamycin, and transformants were grown for 5 days.

### Allelic exchange mutagenesis of the *hp0265* gene in *H. pylori*

To inactivate *hp0265*, a recombinant vector was constructed by a two-step PCR method [27]. The upstream and downstream regions of the *hp0265* gene were amplified from *H. pylori* N6 genomic DNA using two pairs of primers specific for *hp0265* and its flanking regions, N6_HP0265_up_BamHI-N6_HP0265_up_rev1 and N6_HP0265_dw_SalI-N6_HP0265_dw_fwd, respectively. The N6_HP0265_up_rev and N6_HP0265_dw_SalI primers contained nucleotide sequences complementary to each other, respectively. Each PCR product was purified with a Gel extraction kit (A&A Biotechnology). Next, a mixture of two purified products (in equal amounts) was used as a template in a single PCR reaction, using the primers N6_HP0265_up_BamHI-N6_HP0265_dw_fwd. The resulting PCR product contained a 274-nucleotide deletion of *hp0265*. The product was purified and cloned into pBluescript SK II. To add the *aph* gene between the two *hp0265* arms, SmaI restriction enzyme was used to yield suicide plasmid pUWM2019.

Sequence analyses confirmed the correct construction of pUWM2019 and the recombinant plasmid was introduced into *H. pylori* N6 by natural transformation. Cells were plated and screened on plates containing kanamycin and kanamycin with 2 mM DTT to restore the reducing activity of HP0265.

An *hp0265::aph* mutant was obtained by a double cross-over using pUWM2019, and verified by PCR analysis, using the primer pair N6_Hp0264_fwd1-N6_HP0265_rev1.

### Allelic exchange mutagenesis of the *hp0377* gene in *H. pylori*

To inactivate *hp0377*, two recombinant vectors, pUMW529 and pUWM555, were constructed. pUWM529 was generated by a two-step PCR method [27]. Briefly, primers Km1f–Km2r, were used to amplify the *aph* gene from pUWM505. The upstream and downstream regions of the *hp0377* gene were amplified from *H. pylori* N6 genomic DNA using two pairs of primers specific for *hp0377* and its flanking regions, hp0377I-hp0377IIa and hp0377IIIa-hp0377IV, respectively. The hp0377IIa and hp0377IIIa primers contained 5′ leader nucleotide sequences complementary to Km1f and Km2r, respectively [28]. Each PCR product was purified with a Gel extraction kit (A&A Biotechnology). Next a mixture of three purified products (in equal amounts) was used as a template in a single PCR reaction, using primers: hp0377I-hp0377IV. Subsequently, the resulting PCR product, containing the *aph* gene inserted between the two *hp0377* arms in the same transcriptional orientation as the *hp0377* gene, was purified and cloned into pGEM-T Easy, generating the suicide plasmid pUWM529. Sequence analyses confirmed the correct construction of pUWM529, and the recombinant plasmid was introduced into *H. pylori* N6/pUWM509 by natural transformation. Selection was done on blood agar plates containing kanamycin and chloramphenicol, with or without isopropyl-β-d-thiogalactopyranoside.

The pUWM555 plasmid used for insertional mutagenesis of the *H. pylori* N6 *hp0377* gene, was constructed by amplification of the *hp0377* gene and surrounding DNA fragments by PCR, using the hp0377zew1-hp0377zew2 primers. Subsequently, the resulting PCR product was purified and cloned into pGEM-T Easy, creating pUWM543. The resulting construct was digested with BglII, to cut the restriction site in the middle of *hp0377* gene. The sticky ends were blunted using Klenow Fragment. The DNA mixture was purified with a Clean-up kit and ligated with a previously prepared kanamycin cassette, amplified from pUWM505 by the pair of primers Km1f–Km2r. The generated suicide plasmid pUWM555, containing the *aph* gene inserted between the two *hp0377* arms in the same transcriptional orientation as the *hp0377* gene, was confirmed by sequencing. The recombinant plasmid was introduced into *H. pylori* N6/pUWM509 by natural transformation. Selection was done on blood agar plates containing kanamycin and chloramphenicol, with or without isopropyl-β-d-thiogalactopyranoside.

To create a conditional mutant in the *hp0377* gene*,* pUWM509 was constructed based on the shuttle *E. coli*/*H. pylori* plasmid: pILL2150. The *hp0377* coding nucleotide sequence was amplified from *H. pylori* 26695 genomic DNA without its own promoter by the primer pair hp0377mutF - hp0377mutR. The purified PCR products, as well as the shuttle plasmid, were digested with NdeI/BamHI and ligated together to form pUWM509. Correct construction of pUWM509 was confirmed by sequencing. Next, pUWM509 was introduced into *H. pylori* N6, and pUWM529/pUWM555 was used to inactivate *hp0377* gene. The strategy for cloning into pILL2157 was identical.

### Site-directed mutagenesis of the *hp0377* gene

To obtain mutated HP0377 proteins, a set of recombinant plasmids was constructed from pUWM544, which carries the *hp0377* gene without its promoter and signal sequence. Cys-to-Ser point mutations were generated using the Quick Change Site-Directed Mutagenesis Kit (Qiagen) according to the manufacturer’s instructions, starting with 100 ng of pUWM544 template and 125 ng of each primer (primer pairs: N6_377_C89S_for- N6_377_C89S_rev, hp377_C92SII- hp377_C92SII_rev).

To obtain C25A mutated HP0377 protein, a recombinant plasmid was constructed from pUWM399 carrying the *hp0377* gene with its promoter and signal sequence. The Cys-to-Ala point mutation was generated as described above, using primers hp377_C25A_for and hp377_C25A_rev.

### Protein analysis

Preparation of *H. pylori* and *E. coli* protein extracts, SDS-PAGE (sodium dodecyl sulfate polyacrylamide gel electrophoresis) and blotting procedures were performed by standard techniques [26].

### Preparation of subcellular fractions

Subcellular protein fractions were prepared from 48-h *H. pylori* cultures. Periplasmic proteins were released from the cells using an osmotic-shock procedure [29]. After decanting the periplasmic fraction, bacterial pellets were resuspended in 20 mM Tris–HCl, pH 7.5 and sonicated to release the cell contents. Subsequently, cell wall debris was removed and the supernatants were ultracentrifuged (100,000 g, 4 °C, 30 min) to separate the membrane and cytoplasmic fractions. Finally, the cell envelope was fractionated into inner and outer membranes by selective solubilization of the inner membrane with 2.0 % (wt/vol) sodium lauryl sarcosine [30].

### Overexpression and purification of apocytochrome c (HP1227) and HP0231

HP0231 was overexpressed by autoinduction from an *E. coli* Rosetta/pUWM525 strain and purified as previously described [31].

HP1227 expression vector was constructed by amplifying the region encoding the mature HP1227 protein (without the signal sequence, amino acid residues 1–19) from the chromosome of *H. pylori* 26695, with primers Hp1227_NdeI-for and Hp1227_XhoI-rev. The insert was cloned into pET24a with NdeI and XhoI restriction enzymes, to yield plasmid pUWM2090. Expression was induced by 1 mM IPTG (isopropyl β-D-1-thiogalactopyranoside) at OD_600_ ~ 0.6. After 4 h in 37 °C, cultures were centrifuged and the cell pellet was suspended in 50 mM sodium phosphate, pH 8.0, 300 mM NaCl, 10 mM imidazole. Cells were disrupted by ultrasonication. The cell lysate was centrifuged and the resulting supernatant was applied onto Bio-Scale Mini Profinity IMAC Cartridges (Bio-Rad) containing Ni-charged resin. The protein was eluted with an imidazole gradient, using the NGC chromatography system (Bio-Rad).

### Overexpression and purification of HP0377

HP0377 expression vector was constructed by amplifying the region encoding the mature HP0377 protein (without the signal sequence, amino acids 1–24) from the chromosome of *H. pylori* 26695, with primers hp377exI and hp377exII. The insert was cloned into pET28a with NcoI and XhoI restriction enzymes, to yield plasmid pUWM518. The cytoplasm-located HP0377 was overexpressed from pUWM518 by autoinduction [32], and then it was purified by affinity chromatography, dialyzed against Phosphate Buffered Saline (Sigma) and later used for rabbit immunization (Animal Facility, Faculty of Biology, University of Warsaw). The anti-HP0377 rabbit serum was specific and recognized native HP0377, as verified by Western blot analysis.

The HP0377 C89S and C92S expression vectors were constructed by cutting out hp0377-changed sequences from pUWM2045 and pUWM2064, respectively, and cloning the inserts into pET28a as described above, resulting in formation of pUWM2046 and pUWM2065. All plasmids carried the HP0377-His_6_ translation fusion.

For biochemical experiments, the protein was expressed and purified from *E. coli* Rosetta harboring pUWM518, pUWM2046 and pUWM2065. The proteins were overexpressed by autoinduction and then purified by affinity chromatography using NGC Medium-Pressure Chromatography Systems by Bio-Rad as described above.

### Biochemical assays

#### *In vivo* redox state of HP0377

The redox state of HP0377 was visualized by alkylating the free cysteine residues using 4-acetamido-4′-maleimidylstilbene-2,2′-disulfonic acid (AMS, Invitrogen). This agent can only modify covalently free thiols, resulting in a molecular mass increase of 490 Da [31, 33, 34]. Briefly, bacteria were harvested from BA plates after 24 or 48 h of incubation under microaerobic conditions. Samples were standardized using the OD_600_ of the culture, and ice-cold trichloroacetic acid (TCA, final concentration 10 % v/v) was immediately added to the culture. Whole-cell proteins were precipitated and collected by centrifugation, washed with ice-cold acetone, and then dissolved in 50 mM Tris–HCl (pH 7.5), 10 mM ethylenediaminetetraacetic acid (EDTA), 0.1 % SDS containing 20 mM AMS by agitation for 60 min at 37 °C. The proteins in non-reducing Laemmli buffer were resolved by 14 % SDS-PAGE without reducing agent. HP0377 was then detected by an immunoblot analysis using an anti-HP0377 antibody. As controls, we used samples previously treated with 100 mM ditiotreitol (DTT) for 30 min at 30 °C before precipitation of the proteins with TCA.

#### Determination of the p*K*_a_ value

The pH-dependent ionization of the Cys89 and Cys92 was followed by the specific absorbance of the thiolate anion at 240 nm [35]. Measurements were carried out at 25 °C in a buffer consisting of 10 mM Tris, 10 mM sodium citrate, 1 mM EDTA and 200 mM NaCl, pH 11, for EcDsbA as a control, and for HP0377 and its mutated forms, with an average initial protein concentration of 20 μM. The pH of the protein solution was lowered to 2 by the stepwise addition of aliquots of 0.2 M HCl. Absorbances at 240 and 280 nm were recorded on a spectrophotometer and corrected for the volume increase. The pH dependence of the thiolate specific absorbance signal (*S =* (*A*240/*A*280)reduced/(*A*240/*A*280)oxidized) was fitted according to the Henderson-Hasselbach equation.

#### Determination of the redox potential of HP0377

The fractions of reduced and oxidized HP0377 were determined using 4-acetamido-4′-maleimidylstilbene-2,2′-disulfonic acid (AMS) trapping [33]. Briefly, HP0377 (1 μM) were incubated overnight at room temperature in 50 mM KPi pH 7.0, 0.1 mM EDTA and various glutathione (GSH)/glutathione disulfide (GSSG) ratios. After incubation, proteins were precipitated with trichloroacetic acid (TCA) (10 % final concentration). After 20-min incubation on ice, the samples were centrifuged (16,100 × g, 5 min, 4 °C), and the pellets were washed with cold acetone. After a second centrifugation, pellets were dried and resuspended in a buffer containing 20 mM AMS, 0.1 % SDS, 10 mM EDTA, and 50 mM Tris–HCl (pH 7.5). After 45-min incubation at 37 °C, with 1,400 rpm shaking samples were loaded onto 12 % SDS-polyacrylamide gels under denaturing conditions. Fractions of reduced and oxidized protein were determined using ImageJ). The redox potential was then calculated as described previously [36].

#### Alkaline phosphatase (AP) assay

AP activity was measured as previously described [37], with some modifications. Briefly, *E. coli* cell cultures were grown at 37 °C in minimal medium M63 until they reached mid-log phase. Then, the cells were pelleted by centrifugation, washed twice with Tris–HCl (pH 8.0), and resuspended in Tris–HCl (pH 8.0). The OD_600_ was measured spectrophotometrically. Samples (1 ml) were equilibrated for 5 min in a water bath at 28 °C, then 200 μl *p*-nitrophenol phosphate [0.4 % (w/v) in Tris–HCl (pH 8.0)] was added, and the time was recorded. The reaction was allowed to proceed at 28 °C until development of yellow color was observed. At this point the reaction was stopped by adding 200 μl 1 M KH_2_PO_4_. Samples were centrifuged before the *A*_420_ and *A*_550_ measurements.

#### Insulin reduction assay

The ability of HP0377 and EcDsbA to catalyze the reduction of insulin in the presence of DTT was determined as previously described [38].

### Determination of oxidase and isomerase activities

#### Oxidative folding of reduced RNaseA

*In vitro* oxidative folding of reduced RNaseA was performed for HP0377 and EcDsbA as described earlier, with a few modifications [39, 40]. Proteins were oxidized with 50 mM oxidized glutathione (GSSG) and incubated for 1 h at room temperature. RNaseA was reduced by overnight incubation at room temperature in 100 mM Tris acetate pH 8.0 containing 6 M guanidine hydrochloride and 140 mM DTT. All proteins were then dialyzed on desalting columns (Bio-Rad) and concentrated in PBS. Native RNaseA and EcDsbA were used as positive controls. The redox state of the thiols was confirmed by Ellman’s assay, which exploits the colorimetric change at A_412_ when 5,5′-dithiobis-(2-nitrobenzoic acid) (DTNB; ThermoScientific) is converted to 2-nitro-5 thiobenzoate upon cleavage of the disulfide bond by free thiols.

Oxidase activity was measured by analyzing the cleavage of cCMP (Sigma; cytidine 2′:3′-cyclic monophosphate monosodium salt) at A_296_ by refolded RNaseA in the presence of tested enzymes. Reactions (triplicate) were carried out in 200 μl of PBS buffer containing 100 mM Tris acetate pH 8.0, 2 mM EDTA, 0.2 mM GSSG, 1 mM GSH (reduced glutathione), 4.5 mM cCMP, RNaseA (10 μM) and analyzed enzyme (20 μM). The reaction mixtures were prepared in a 96-well plate format and read through 30 min at 27 °C in a Sunrise™ (Tecan) plate reader. Three independent experiments were performed.

#### Refolding of scrambled RNaseA

*In vitro* refolding of scrambled RNaseA was performed for HP0377 and EcDsbC as described earlier, with a few modifications [41, 42, 39]. Proteins were reduced with 100 mM DTT and incubated overnight at 4 °C. RNaseA was first reduced by overnight incubation at room temperature in 100 mM Tris acetate pH 8.0 containing 6 M guanidine hydrochloride and 140 mM DTT. Then, in order to introduce incorrect disulfides, reduced RNaseA was dialyzed against PBS buffer containing 6 M guanidine hydrochloride, sparged with oxygen and incubated for 3 days in the dark at room temperature. Finally, 2 mM hydrogen peroxide (Sigma) was added for 30 min at 25 °C. All proteins were then dialyzed on desalting columns (Bio-Rad) and concentrated in PBS. EcDsbC was used as a positive control. The redox state of the thiols was confirmed by Ellman’s assay.

RNaseA activity was measured by analyzing the cleavage of cCMP as described for the oxidative test, with a change in the reaction mixture: 100 mM Tris acetate pH 8.0, 2 mM EDTA, 10 μM DTT, 4.5 mM cCMP, RNaseA (40 μM) and analyzed enzyme (20 μM). Three independent experiments were performed.

#### Determination of the interaction between HP0231 and HP1227

Oxidized HP0231 was prepared by incubating the purified HP0231 with a 100 mM oxidized glutathione for 1 h at 37 °C. Reduced apocytochrome c was prepared by incubating the purified apocytochrome with 10 mM DTT for 1 h at 37 °C. Excess DTT and oxidized glutathione was removed using desalting columns that were previously equilibrated with 20 mM Tris HCl pH 7.9, 150 mM NaCl. Reduced apocytochrome c in 20 mM Tris–HCl pH 7.9, 150 mM NaCl was incubated for 3 h at 37 °C in the presence of a twofold excess of HP0231_ox_. After incubation, the proteins were precipitated with trichloroacetic acid [final concentration 10 % (w/v)], washed three times with ice-cold acetone and then resuspended in 100 μl of a reaction buffer consisting of 50 mM Tris–HCl pH 6.8, 2 % (w/v) SDS, 10 mM AMS. The oxidized and reduced forms of HP0231 as well as reduced apocytochrome c were similarly treated with AMS before SDS–PAGE under nonreducing conditions. AMS-treated protein samples were separated by nonreducing SDS–PAGE and visualized by Coomassie staining.

#### Size exclusion chromatography

To verify that HP0377 exists as a monomeric protein, size exclusion chromatography was employed. HP0377 was loaded onto Superdex75 HiLoad 16/600 column (GE Healthcare) and eluted with 20 mM Tris pH 8, 150 mM NaCl.

#### Determination of the oligomeric state of HP0377 using glutaraldehyde

Crosslinking of polypeptide chains with glutaraldehyde was performed essentially as described [43].

HP0377 (2.5 mg/ml) was incubated at room temperature with different concentration of glutaraldehyde (0.001-0.1 % v/v) in 0.05 M bicine–NaOH buffer (pH 8.5), 0.1 mM DTT, 0.4 M NaCl for 20 min and the reaction was quenched by adding ethanolamine–HCl (pH 8.0) to a final concentration of 0.14 M.

### Phenotype assays

#### DTT sensitivity assay

DTT sensitivity experiments were performed as previously described [40]. A freshly prepared 1 M DTT (Applichem) stock solution was dissolved in molten LB agar to the final concentration of 12 mM DTT. The DTT agar plates were used within 30 min of pouring to prevent oxidation of DTT by air. Exponentially growing cultures were decimally diluted, and 7 μl aliquots were spotted on the plates. The growth was observed after overnight incubation at 37 °C. The experiments were conducted in triplicate.

#### Motility assays

*E. coli* cells were grown in liquid culture in LB broth until the OD_600_ value was close to 1. Then bacteria were inoculated on LB soft agar plates containing 0.35 % (w/v) agar with a sterile toothpick and incubated for 18 h at 30 °C.

#### Copper sensitivity assay

The copper sensitivity assay was performed as previously described [31]. Briefly, bacteria were grown in BHI media supplemented with 8 mM or 10 mM CuCl_2_. Strains were grown at 34 °C.

### Ethics statement

All studies involving animals were performed in accordance with ethical standards, after approval from the Local Ethics Committee No. 1, Warsaw, Poland 966/2009.

## Results

### Determination of the *in vivo* redox state of HP0377

HP0377 is an oxidoreductase, as it contains the TRX domain with a CXXC (CSYC) motif located at the N-terminus of the first helix in the TRX fold. The involvement of HP0377 in the cytochrome c maturation process is indicated by its structure, its *in vitro* interaction with HP1227 (cytochrome c_553_) and the genetic organization of the DNA fragment containing the *hp0377* gene [20, 22].

To clearly define whether HP0377 functions in the oxidizing pathway or the reducing pathway, we first decided to determine the redox state of that protein *in vivo* using the AMS-trapping technique [33]. The *in vivo* redox state of an oxidoreductase usually reflects its activity in the cell, i.e., proteins that function in the oxidizing pathway, such as EcDsbA, are maintained in the oxidized state *in vivo*, whereas proteins that function in the reducing pathway, such as CcmG, are maintained predominantly in the reduced state. We found that HP0377 is present in the reduced form in wild-type cells, which suggests that HP0377 functions as a reductase in *H. pylori,* which is in accord with its three dimensional structure [22].

**Fig. 1:**
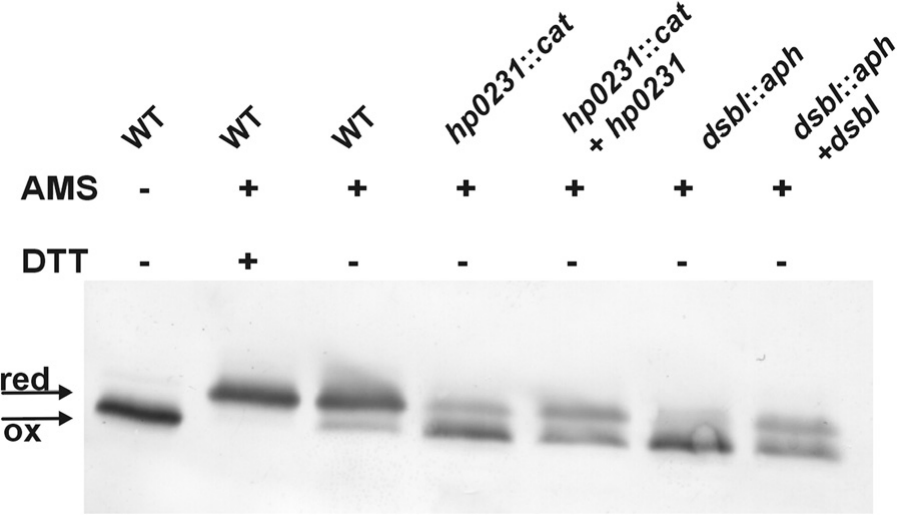
Redox state of HP0377 in wt and mutants: *hp0231*::*cat*, *dsbI*::*aph* and complemented strains of *H. pylori* strain N6. Bacterial cultures were treated with 10 % TCA, followed by alkylation with AMS. Cellular proteins including the reduced (red; DTT treated, modified with AMS) and the oxidized (ox; non-modified with AMS) controls were separated by 14 % SDS-PAGE under non-reducing conditions, and Western blot analysis antibodies against HP0377 was performed. Each lane contains proteins isolated from the same amount of bacteria

**Fig. 2:**
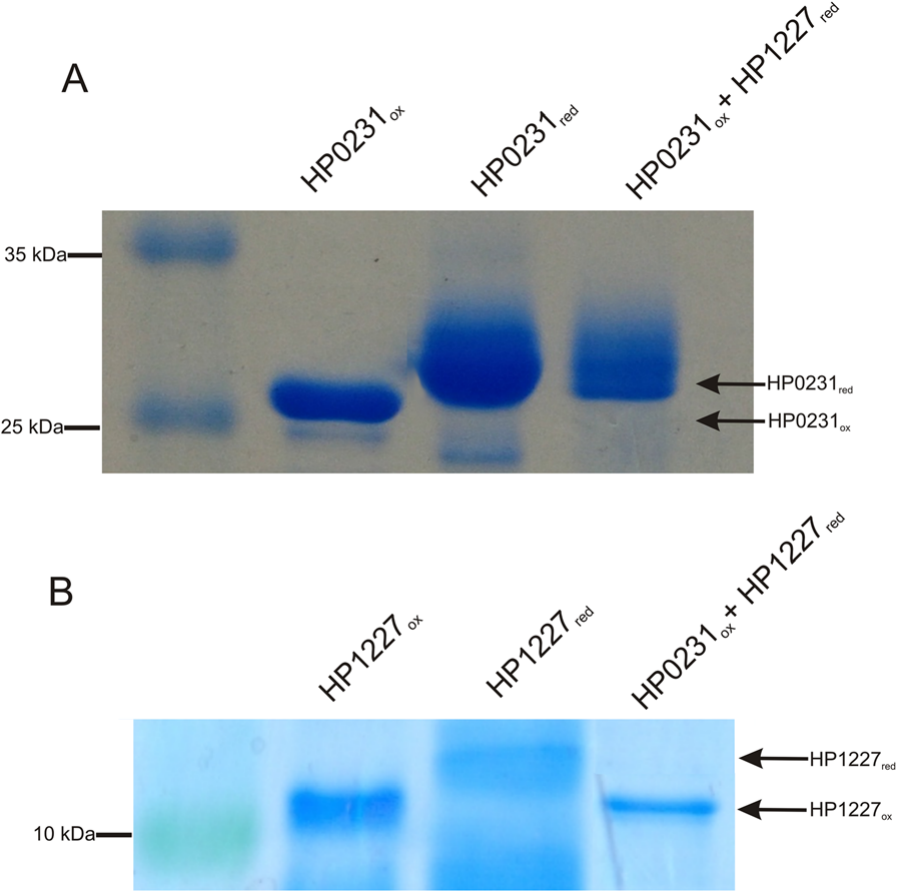
Oxidase assay of HP0231 towards apocytochrome c (HP1227)*.* Two different SDS PAGEs were run to better visualize the shift between oxidized and reduced forms of proteins. **a** HP0231-dependent oxidation of reduced apocytochrome c. Lane 1, oxidized HP0231. Lane 2, reduced HP0231, Lane 3, reduced apocytochrome c treated with oxidized HP0231. **b** HP0231-dependent oxidation of reduced apocytochrome c. Lane 1, oxidized apocytochrome c. Lane 2, reduced apocytochrome c, Lane 3, reduced apocytochrome c treated with oxidized HP0231. Different redox forms were detected by nonreducing SDS-PAGE after AMS treatment, which results in an increase in the molecular mass of reduced proteins by about 0.5 kDa per thiol group

Thus, we also determined the redox status of the HP0377 in *H. pylori* lacking HP0231 or DsbI (HP0595). Both proteins are active in the Dsb oxidative pathway. As described earlier, HP0231 introduces disulfide bonds and DsbI is partially responsible for HP0231 re-oxidation [31]. Our results showed that a significant portion of HP0377 is present in the oxidized form in both the *hp0231* and *dsbI* mutated cells (Fig. 1). Also the overproduction of HP0231 or DsbI from a moderate copy number plasmid disturbs the redox homeostasis and results in the presence of HP0377 in both reduced and oxidized forms (Fig. 1). To clarify the link between HP0231 and cytochrome c biogenesis, we decided to check whether apocytochrome c is a substrate of HP0231. We found that HP0231 was able to oxidize the reduced apocytochrome c *in vitro* (Fig. 2)

.

### Biochemical characterization of HP0377

To clearly define whether HP0377 functions as oxidoreductase, we analyzed the biochemical properties of the protein. Recombinant proteins used for biochemical analysis were produced as cytoplasmic proteins in *E. coli* cells and purified by affinity chromatography. First, to gain insight into the role played by HP0377, we determined its redox potential by equilibrium incubation using GSH/GSSG as a reference. We found that the HP0377 redox potential is – 171 mV (Additional file 1: Figure S1), which makes it a rather weak reductant.

**Fig. 3:**
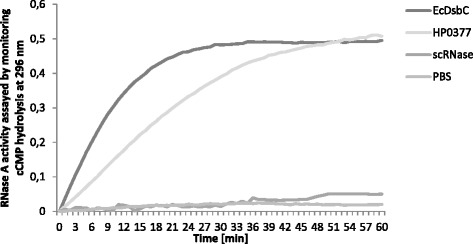
Isomerase activity assay. The reaction contained 40 μM scrambled RNase in 200 mM potassium phosphate buffer, pH 7.0, 2 mM EDTA, 20 μM DTT, and 9 mM cCMP. The reaction was performed in the absence or presence of 20 μM EcDsbC, 20 μM HP0377. The cleavage of cCMP by refolded RNase was monitored continuously at 296 nm. The changes in the absorbance at 296 nm as a function of time are presented. Three independent experiments were performed

**Fig. 4:**
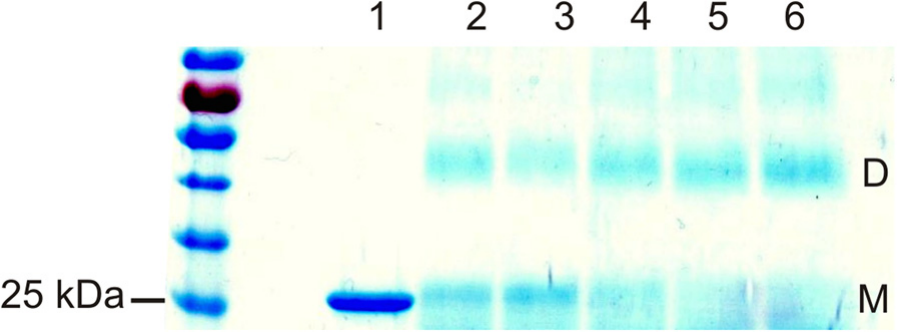
Glutaraldehyde crosslinking of HP0377. Purified HP0377 at 2.5 mg/ml was cross-linked in the presence of different concentration of glutaraldehyde: lane 1–purified HP0377 protein, lane 2–0.001 %, lane 3–0.005 %, lane 4–0.01 %, lane 5–0.05 %, lane 6–0.1 % glutaraldehyde. M–monomers, D–dimers

**Fig. 5:**
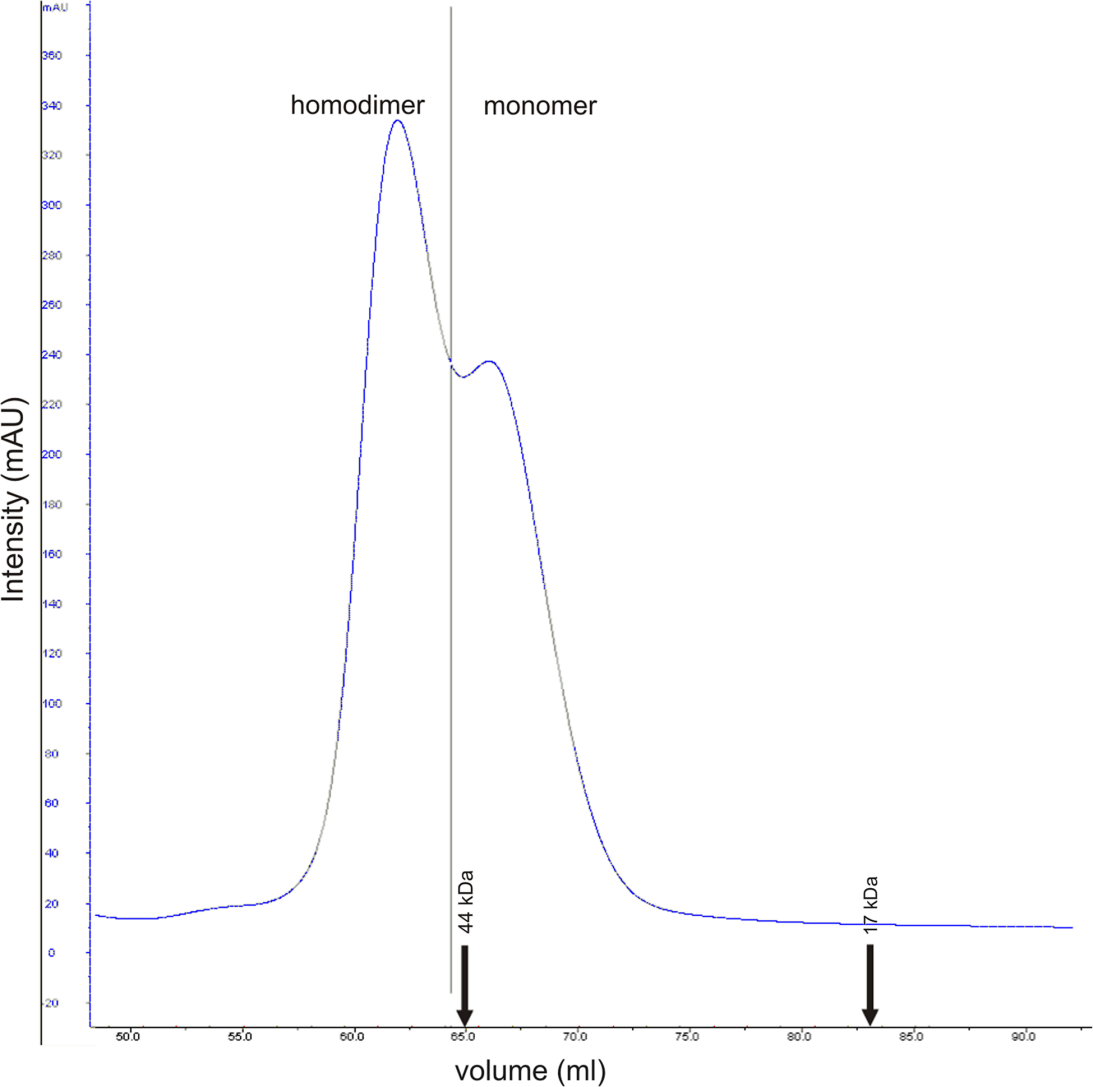
Size exclusion profile of the purified HP0377 separated on a Superdex75 HiLoad 16/600 column (GE Healthcare) and monitored by absorbance at 280 nm. HP0377 elutes as a two peaks at 61 min, with an estimated mass of 48 kDa, consistent with the size of the homodimer and 66 min. with estimated mass of 24 kDa, consistent with the size of the monomer. The column was calibrated with Gel Filtration Standard (Bio-Rad): Thyroglobulin (670 kDa), γ-globulin (158 kDa), Ovalbumin (44 kDa), Myoglobin (17 kDa), Vitamin B12 (1.35 kDa). The relative positions of the chosen standards are marked with arrows

Next we determined the ability of HP0377 to reduce insulin in the presence of DTT. The insulin reduction assay is commonly used to determine whether a protein can function as an oxidoreductase, regardless of its function in the reducing or the oxidizing pathway *in vivo*. Insulin contains two intramolecular disulfide bonds that connect the A and B chains: reduction of these disulfide bonds causes the precipitation of the B chain, which can be monitored by following the increase of turbidity at 650 nm [38]. As shown in Additional file 2: Figure S2, the reaction lag time with HP0377 in the insulin reduction assay is almost as long as the control reaction (about 60 min), whereas the lag time with EcDsbA is 24 min. Therefore, HP0377 likely cooperates with a specific substrate, which is typical of CcmG proteins. However, these data are in contrast to some previous data [22]. To clarify this inconsistency, and because there is a similarity between the active site of HP0377 (CSYC motif and Thr in cis-Pro loop) and the disulfide isomerase EcDsbC (CPYC motif and Thr in cis-Pro loop), we performed a disulfide isomerase assay by evaluating the ability of HP0377 to reactivate oxidized, scrambled RNase (scRNaseA). ScRNase was prepared as described in the methods section and did not contain free cysteines, as confirmed with Ellman reagent. In this assay, the refolding efficiency of HP0377 was almost as high as EcDsbC (Fig. 3). To get a complete biochemical characterization of HP0377, we also investigated its ability to catalyze the refolding of reduced-unfolded RNaseA. As expected, HP0377 did not show activity in this assay (Additional file 3: Figure S3). As HP0377 revealed high isomerizing activity, and because Dsb proteins involved in the isomerization pathway exist as dimers, we also evaluated the potential oligomerization of HP0377 using two methods (gel filtration and glutaraldehyde crosslinking strategy) (Figs. 4, 5) HP0377 that lacked its own signal sequence (the 25-221 amino acid residues of the native HP0377) and contained a C-terminal 6 His tag was purified from *E. coli* cytoplasm and used in both assays. We found that exposure of HP0377 to glutaraldehyde, which stabilizes oligomeric proteins by covalent crosslink formation, resulted in generation of a protein with a molecular weight of 48 kDa. This result clearly showed that HP0377 can exist as a dimer. The size exclusion experiment showed that HP0377 eluted as two peaks, one with an estimated mass of 24 kDa, consistent with the size of the monomer, and the second with estimated mass of 48 kDa, consistent with the size of the homodimer. Thus, the presented data allowed us to conclude that at least a portion of HP0377 exists as a dimer.

**Fig. 6:**
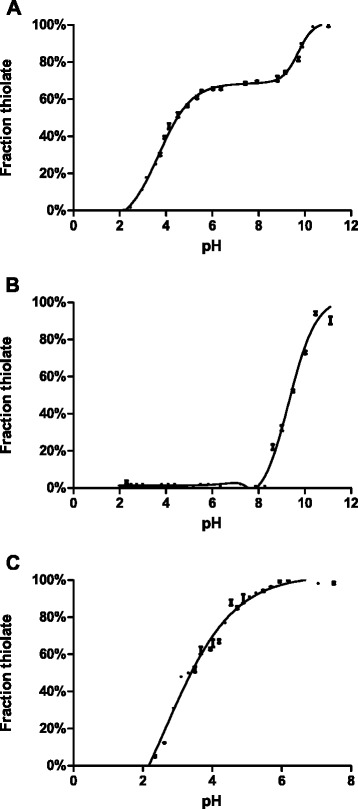
Thiol ionization equilibrium in HP0377 followed by UV absorbance at 240 nm. Panel **a**: HP0377 wild type. Panel **b**: HP0377 C89S. Panel **c**: HP0377 C92S

The function of thiol-oxidoreductases depends on the p*K*_a_ values of their active-site Cys residues. The pKa of the HP0377 active site thiols was determined by observing the change in absorption of the thiolate anion at 240 nm as a function of pH in wt protein. The pH titration of wt HP0377 showed two transitions, one with p*K*_a_ = 3.56 ± 0.11 and the other with p*K*_a_ = 9.23 ± 0.21 Fig. 6a. Because these data are atypical for CcmGs, we constructed two single-Cys CXXC motif mutants of HP0377 (C89S and C92S), which allowed independent measurement of the p*K*_a_ of each Cys residue. As shown in Fig. 6b, c the C89S mutant had a p*K*_a_ = 3.46 ± 0.24 and the C92S mutant a p*K*_a_ = 9.41 ± 0.15. These results agreed with the titrations of the wt protein. The p*K*_a_ value of the solvent-exposed active site cysteine (Cys89) is about 3.5. This is relatively acidic compared with the solvent-exposed active site cysteine of the *E. coli* CcmG protein, p*K*_a_ = 6.8 [44], or the *B. subtilis* ResA, p*K*_a_ = 8.8 [45]; it is as acidic as *E. coli* DsbA, which is a known oxidant whose p*K*_a_ of the solvent-exposed active site cysteine is 3.5 [38], and it is close to the p*K*_a_ of EcDsbC, 4.1 ± 0.3 [46]. Thus, HP0377 is the first described CcmG protein having an acidic p*K*_a_ of the N-terminal cysteine of the CXXC motif, and at the same time, it presents a low redox potential.

### HP0377 is an essential protein for *H. pylori*

To investigate the function of HP0377 in *H. pylori* we decided to construct an isogenic *hp0377* knock-out strain by allelic exchange. Initial attempts to knock out *hp0377* using kanamycin or chloramphenicol antibiotic cassettes and the allelic exchange methodology failed. Several laboratories have reported that loss of active CcmG can be compensated by the addition of reducing agents to the growth medium. Therefore, we tested whether the addition of the DTT into growth medium enable us to obtain an *H. pylor*i N6/26695 *hp0377* mutated strain. It did not. Moreover, our attempts at trans-complementation of a genomic deletion with a plasmid-borne copy of *hp0377*, based on the strategy using modified pHeL2 complementing plasmids of low and high copy number described by Boneca et al. were also unsuccessful [47].

Taken together, these studies, in combination with our observation that a *H. pylori hp0265* mutated strain can only be generated in the presence of a reducing agent, suggest that both HP0377 and HP0378 provide an essential function in *H. pylori*.

### HP0265 is a redox partner of HP0377

**Fig. 7:**
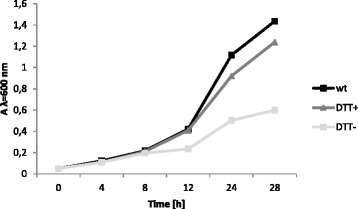
Effect of DTT on *Helicobacter pylori hp0265* ^*−*^ growth. Cultures of *H. pylori hp0265* ^*−*^ were grown in Brain Heart Infusion (BHI) broth supplemented with 10 % fetal bovine serum and kanamycin. One culture was additionally supplemented with 2 mM DTT. As a control wild type of *Helicobacter pylori* N6 was grown under the same conditions

**Fig. 8:**
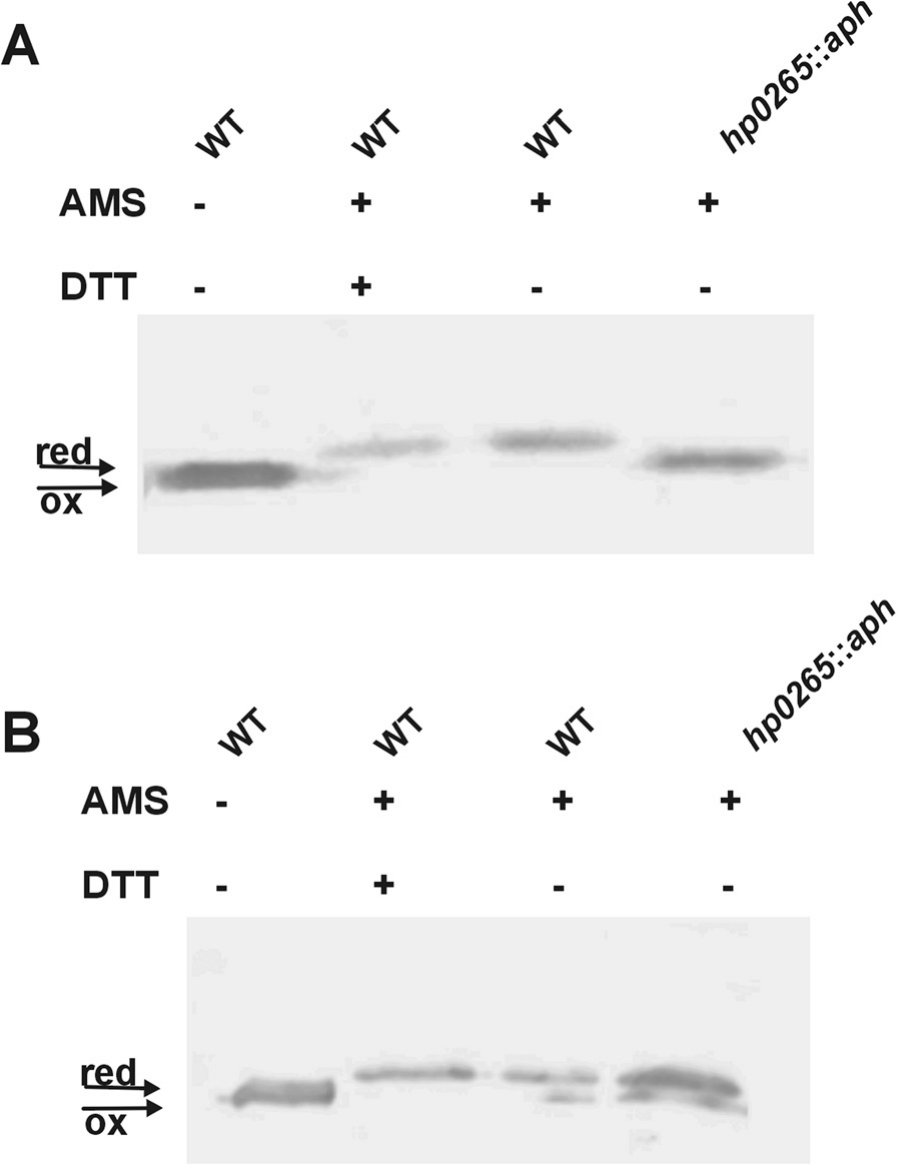
Redox state of HP0377 in wt and mutant: *hp0265*::*aph* measured for cells grown in the absence (**a**) or presence (**b**) of DTT. Bacterial cultures were treated with 10 % TCA, followed by alkylation with AMS. Cellular proteins including the reduced (red; DTT treated, modified with AMS) and the oxidized (ox; non-modified with AMS) controls were separated by 14 % SDS-PAGE under non-reducing conditions, and Western blot analysis using antibodies against HP0377 was performed. Each lane contains proteins isolated from the same amount of bacteria

Dsb proteins that play a role in a cytochrome-c biogenesis, in both system I and system II, are kept in the reduced form by the integral membrane protein DsbD, or its shortened analogue CcdA. Both proteins catalyze the transfer of electrons from cytoplasmic thioredoxin across the inner membrane to the periplasm; however the mechanism of the process in the case of CcdA is still unknown [48]. Apart from HP0265, which was described as CcdA, a search for Dsb homologs in the *H. pylori* genome, employing the *E. coli* Dsb sequence as a query sequence, revealed that *H. pylori* contains another DsbD homolog (i.e., HP0861). Similar to *Rodobacter capsulatus* CcdA and *Bacillus subtilis* CcdA, HP0265 is a six-transmembrane (TM) protein containing two cysteines at the end of the first and the fourth TM segments [48]. HP0861, however, has five cysteines with an unusual, and as yet uncharacterized, spatial arrangement, and it could be involved in a process other than the cytochrome c biogenesis. Thus, to establish whether HP0265 is responsible for re-reducing HP0377, we created a *H. pylori* strain lacking *hp0265*. The recombinant plasmid pUWM2019 (based on a vector non-replicating in *Helicobacter* cells) was used for the mutagenesis. The plasmid contains the *hp0265* gene disrupted by insertion of a kanamycin resistance cassette into the gene coding sequence. The *hp0265* mutants were only produced in the presence of the reducing agent DTT, which indicates the importance of HP0265 and its reducing activity for bacterial survival. We then tested whether deletion of *hp0265* affects the redox state of HP0377. We noticed that growth of the *hp0265* mutated strain without DTT was much slower than growth of the wt strain (Fig. 7). Therefore, to check the influence of HP0265 on the HP0377 redox state, the *H. pylori* N6 *hp0265*^*−*^ strain was cultivated 24 h longer than the wt strain to achieve the same cell density. As shown in Fig. 8a, the knock-out of *hp0265* results in the accumulation of a noticeable amount of HP0377 in the oxidized state, which indicates the role of HP0265 in re-reducing HP0377. The addition of DTT to the medium resulted in the presence of HP0377 in the reduced form, clearly verifying the role of HP0265 (Fig. 8b).

### Localization of HP0377

**Fig. 9:**
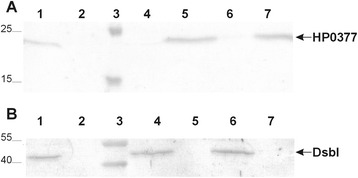
Localization of HP0377. Panel **a**: Immunoblot analysis with subcellular fractions of *H. pylori* strain 26695 using anti-HP0377 antibody. Lanes: 1 - whole cell lysate; 2 - periplasmic proteins; 3 - protein ladder; 4 - cytoplasmic proteins, 5 - inner membrane proteins; 6 - outer membrane proteins; 7 - membranes protein. Panel **b**: Immunoblot analysis with subcellular fractions of *H. pylori* strain 26695 using anti-DsbI antibody. Lanes: 1 - whole cell lysate; 2 - periplasmic proteins; 3 - protein ladder; 4 - inner membrane proteins; 5 - outer membrane proteins; 6 - membrane proteins, 7 - cytoplasmic proteins

Most of the CcmG proteins are membrane-anchored via the N-terminal transmembrane domain. *In silico* analysis (HMM Expasy, TOPCON, Smart programs) did not indicate the presence of the transmembrane N-terminal domain in HP0377, though it confirmed the occurrence of this kind of domain located in the N-terminus of *B. subtilis* ResA. At the same time, *in silico* analysis using the LipoP 1.0 server showed that the HP0377 amino-acid sequence contains a putative signal sequence that can be processed by signal peptidase II, which suggested that HP0377 is an inner membrane lipoprotein. To confirm the predicted localization of HP0377 in *H. pylori*, a subcellular fractionation experiment was carried out. Detection of HP0377 with specific rabbit antibody against rHP0377 revealed that HP0377 is present in the inner membrane proteins compartment (Fig. 9a). As a control for the method of subcellular fractionation of *H. pylori* used, we traced the cellular location of DsbI (i.e., HP0595), which is an inner-membrane protein (Fig. 9b).

HP0377 contains an LSAC motif located in the C-terminal of its potential signal sequence. It has been shown that the two main strategies commonly used to confirm lipid modification of a protein (radiolabeling with palmitic acid and inhibition of the signal peptidase II activity by globomycin) have not worked in *H. pylori* [49, 50]. Thus, to provide further insight into the role of LSAC motif of HP0377, we carried out alanine mutagenesis of C25. A gene encoding the C25A mutated protein was cloned into pHel2 and introduced into *H. pylori* N6. In this experiment, we used the wt strain as we had not been able to generate a knock-out of *hp0377.* We expected that the mutated form of the HP0377 (C25A), overexpressed from the plasmid, would locate in the periplasm or cytoplasm. We found that the overexpression of HP0377 had no effect on cell growth and that the changing C25 into A in the LSAC motif of HP0377 did not influence its anchoring to the cytoplasmic membrane (data not shown).

### HP0377 is not active in *E. coli* cells

To test whether it would be possible to study *hp0377* activity in *E. coli* cells, the *hp0377* gene was cloned with its own promoter into a shuttle plasmid, pHEL2 (pUWM399), and introduced by transformation into wild-type *E. coli*, and into *E. coli dsbA* and *E. coli dsbC* mutated cells. The presence of HP0377 protein expressed from recombinant plasmid in *E. coli* cells was confirmed by Western blot analysis using specific rabbit anti-HP0377 serum (Additional file 4: Figure S4). We found that HP0377 does not restore the *dsbA* wild type phenotype, as measured by motility and DTT tests, as well as by the alkaline phosphatase assay (Additional file 5: Figure S5). Moreover, HP0377 is not able to complement the *E. colidsbC* mutant, as measured by the copper sensitivity assay (Additional file 6: Figure S6).

**Fig. 10:**
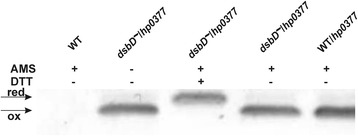
Redox state of HP0377 in wt and *dsbD* mutant *E. coli* strain transformed with pUWM399 carrying *hp0377*. Bacterial cultures were treated with 10 % TCA, followed by alkylation with AMS. Cellular proteins including the reduced (red; DTT treated, modified with AMS) and the oxidized (ox; non-modified with AMS) controls were separated by 14 % SDS-PAGE under non-reducing conditions, and Western blot analysis using antibodies against HP0377 was performed. Each lane contains proteins isolated from the same amount of bacteria

To clarify the lack of complementation of the *EcdsbC* mutation by HP0377, we determined its redox state in *E. coli* cells. In both the *E. coli* wild type and in *dsbD* mutated cells, HP0377 exists in an oxidized form, in contrast to what was observed in *H. pylori,* where it was present in a reduced form (Fig. 10). These results suggest that HP0377 does not cooperate with *E. coli* DsbD.

The *H. pylori* genome does not contain a *dsbD* gene, and HP0377 is reduced by CcdA (HP0265). Thus, to start analysis of the HP0377 function in an *E. coli* background, we attempted to introduce *hp0265* into *E. coli*. We cloned *hp0265* into the low copy number plasmid pMPM-K6Ω, which has an arabinose inducible promoter. After the correctness of the recombinant plasmid construction was verified by sequencing, the plasmid was introduced by transformation into *wt E. coli* and a *dsbD* mutant *E. coli* that harbored a plasmid expressing HP0377. When the expression of *hp0265* was induced by 0.2 % arabinose, the production of HP0265 was lethal for cells (Additional file 7: Figure S7).

## Discussion

**Fig. 11:**
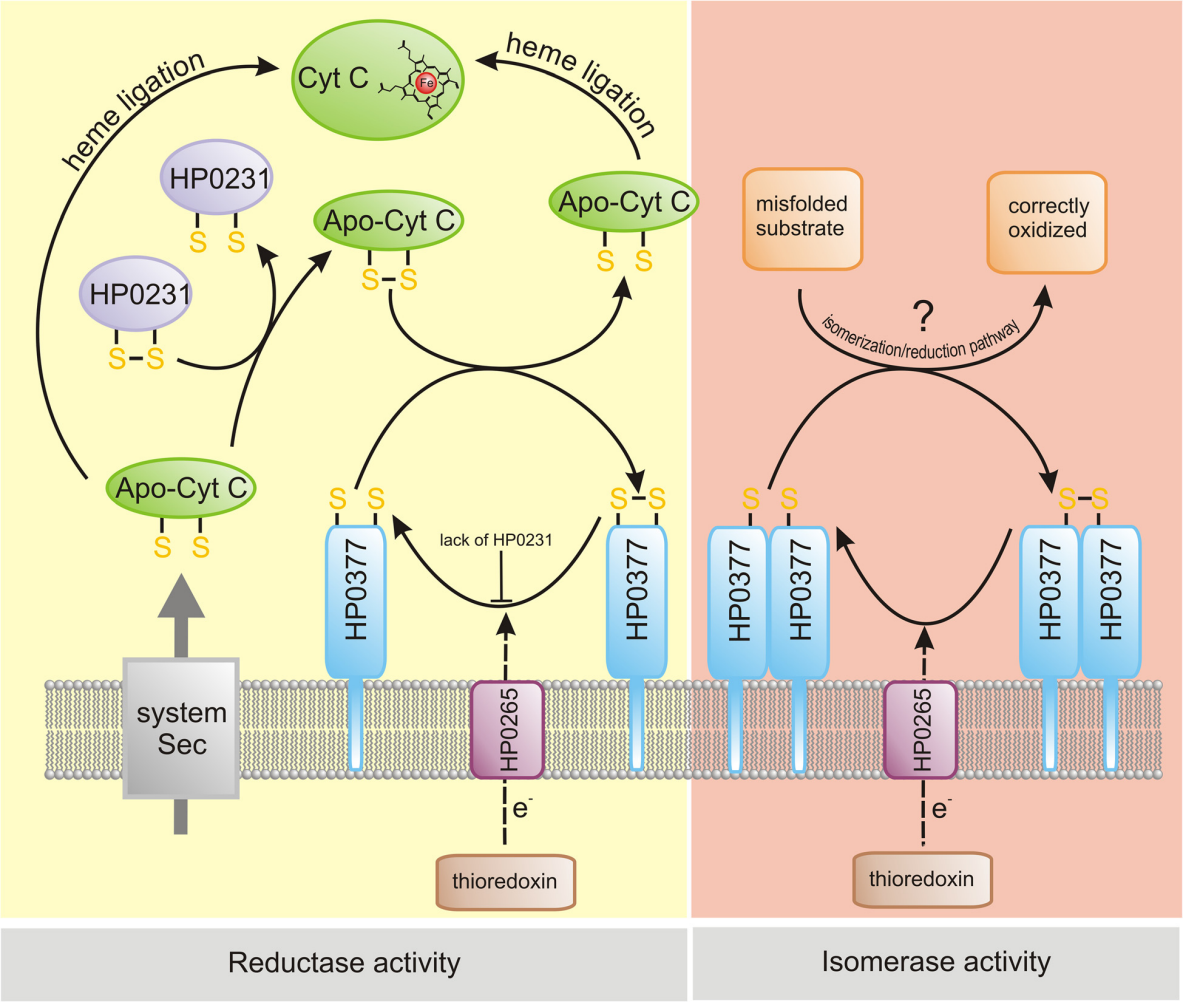
Models representing the role of *H. pylori* HP0377 in cytochrome c biogenesis and in oxidized protein folding. Following synthesis, apocytochrome c is translocated across the membrane by the Sec protein complex, and there it is oxidized by HP0231 (periplasmic dimeric oxidoreductase). Subsequently, its CXXCH motif is reduced by HP0377, and it is ligated to heme. Likely, as observed for other microorganisms, some molecules of the apocytochrome c are not oxidized and are directly ligated with heme (HP0231-independent pathway). Lack of HP0231 prevents HP0377 reduction by HP0265. A small portion of HP0377 is present in a dimeric form and potentially plays a role in the Dsb isomerization/reduction pathway. So far, the substrates of this pathway remains unknown

HP0377 is a thioredoxin-fold protein containing the CSYC motif, which indicates that it functions as a disulfide oxidoreductase. Although there is no evidence that HP0377 is involved in cytochrome c assembly *in vivo*, that is the likely case because its resolved structure is similar to that of other CcmG proteins, and because it is able to reduce the oxidized form of apocytochrome c *in vitro* [23]. Additionally, it is co-transcribed with the *ccsBA* (*hp0378*) gene that is involved in heme transport and its ligation to apocytochrome c [20, 21]. In this work we have showed that HP0377 is present *in vivo* in the reduced form, which is a characteristic feature of thiol oxidoreductases being reductants. The HP0377 redox state *in vivo* is consistent with its redox potential determined by us (-171 mV) and by others (-180 mV), which classifies HP0377 as a mild reductant. This value is in good accord with the standard redox potential determined for the most gram-negative CcmG proteins, such as EcCcmG (-178 mV), BjCcmG (-217 mV) or PaCcmG (-213 mV) [51–53]. Additionally, we have shown that HP0377, like other CcmGs, does not reduce insulin. This finding is consistent with a generally accepted view that CcmG proteins are specific thiol-oxidoreductases involved in only the cytochrome c maturation pathway. So far, the only known exception to this rule is TlpA from *B. japonicum*. This protein is a reductant for the copper metallochapherone Scol, but it also acts in the cytochrome maturation process and catalyzes the insulin reduction [54]. The TlpA structure, when compared to other CcmGs, revealed some unusual properties that potentially substantiate its activity in the insulin reduction assay [55, 56]. Although HP0377 is inactive in the insulin reduction assay, it confers a disulfide isomerase activity almost as high as that of EcDsbC. This atypical HP0377 attribute is in accord with its capability to generate a dimeric form, as shown by size exclusion and glutaradehyde crosslinking methods. It should be noted, in contrast to our data, that Yoon et al. reported that HP0377 is monomeric. As we and Yoon et al. used almost identical recombinant HP0377 proteins, the inconsistency may result from various experimental methods employed [22]. Furthermore, the p*K*_a_ of the N-terminal cysteine of the CXXC motif of HP0377 appears to be similar to those observed for EcDsbC or EcDsbA but not to those determined for most CcmGs. This observation suggests that the activity of HP0377 in *H. pylori,* which possesses only two proteins having a TRX fold with the CXXC motif (i.e., HP0231 and HP0377), is distinct from that described for the classical CcmGs. Taking into account that there is no classical DsbC protein in the *H. pylori* proteome and the dimeric HP0231 does not catalyze the recovery of active RNase from scrambled RNase (unpublished data), we concluded that HP0377, in contrast to most CcmGs that are involved in only the cytochrome c biogenesis process, is at least a bifunctional reductase. Figure 11 presents a model of HP0377 functioning. As the gel filtration experiment indicated that HP0377 exists as a mixture of monomeric and dimeric forms, its dual function may be regulated by the mutual ratio between the two forms. This suggestion is also supported by our interesting finding that HP0377 is essential for cell viability. Additionally, the *in vivo* experiments provide some intriguing data. We have noticed that the *in vivo* redox state of HP0377 is conditioned by the presence of HP0231 or HP0595. Previous experiments on the function of HP0231 showed that it is a periplasmic oxidase, and that HP0595 is partially responsible for HP0231 reoxidation. Therefore, they create a redox pair playing a role in introduction of disulfide bonds [31]. As *H. pylori* does not encode a classical DsbA, HP0231 may oxidize apocytochrome c just after its transport across the inner membrane. By analogy to the function of *B. subtilis* ResA, we suggest that lack of oxidized apocytochrome c in *hp0231* mutated cells prevents HP0377 from reacting with its redox partner (i.e., HP0265), so a part of HP0377 remains in the oxidized form [57]. However this hypothesis requires verification because HP0377, as indicated by biochemical characterization, may also play a role in processes other than the cytochrome c maturation process. Overproduction of the periplasmic thiol oxidases, HP0231 and HP0595, results in changes to the mutual ratio of the reduced and oxidized forms of HP0377. As in the *hp0231* mutated cells, only part of HP0377 is present in a reduced form. Since the presence of this form is necessary for HP0377 to fulfill its function, we decided to compare the cytochrome c activity in *hp0231* mutated cells to that observed in *wt* cells, using the TMPD-oxidation assay. TMPD (N,N,N′,N′-Tetramethyl-p-phenylenediamine) is an artificial substrate of cytochrome c oxidase [58]. We did not observe any differences in bacterial cell pigmentation (data not shown), indicating that when the main periplasmic oxidase, i.e., HP0231, is absent, full HP0377 activity is probably dispensable for cytochrome c biogenesis.

Most of the gram-negative bacteria CcmGs are membrane-anchored, periplasm-facing proteins [2]. We also tested the subcellular localization of HP0377 by cell fractionation experiments, which showed that it is present in the inner membrane proteins fraction. These data contradict those of Yoon et al., who classified HP0377 as periplasmic protein [22]. HP0377 contains a putative signal sequence with a lipobox (LSAC) that can potentially be processed by signal peptidase II, which is specific for lipoprotein precursors [59]. It has been shown that a lipoprotein signal peptidase gene (*hp0074*) is present in the *H. pylori* genome and that it is essential for this bacterium [28]. However current knowledge about the mechanism for lipoproteins transport across the inner membrane and their sorting in *H. pylori* is still limited and comes mainly from bioinformatic studies [60]. We hypothesize that HP0377 is a lipoprotein similar to two well-characterized *H. pylori* lipoproteins, Lpp20 and HpaA, which also contain a lipobox (LVGS) and show inhibited processing, at least when expressed in *E. coli* cells, by a globomicin, a cyclic peptide antibiotic specific for signal peptidase II [49, 50]. Unexpectedly, changing the C of the LSAC HP0377 motif into A did not influence its cell localization. Understanding the role of the HP0377 lipobox requires more investigation, as has been done for some *Legionella* lipoproteins [61].

CcmG proteins that play a role in a cytochrome c biogenesis, in both system I and system II, are kept in the reduced form by the integral membrane protein DsbD or its shorter analogue, CcdA. Both proteins catalyze the transfer of electrons from cytoplasmic thioredoxin across the inner membrane to the periplasm. DsbD consists of eight transmembrane segments (β domain), an N-terminal (α domain) and a C-terminal domain (γ domain). Both the N- and C-terminal domains face the periplasm [62, 63]. CcdA, which is a shorter version of DsbD, consist of only the β transmembrane domain of DsbD. In contrast to DsbD, which transfers reducing potential to a large number of periplasmic proteins, CcdA was thought to be only involved in the cytochrome c maturation process [48, 64]. However, recently published data, have showed that in *Bacillus subtilis* or *B. anthracis* CcdA plays a role also in sporulation and virulence [65, 66]. Recently a new class of DsbD proteins, named ScsB – which have a domain organization similar to but not identical with that of DsbD – has been described. However, their role in CcmG re-reduction has not yet been analyzed [67]. We show that HP0377 is kept in the reduced form by HP0265 and does not cooperate with *E. coli* EcDsbD. In contrast to our data, it was demonstrated by Katzen et al. that CcdA from *Rhodobacter capsulatus* complements an *E. coli dsbD* gene deletion, and EcDsbD complements the lack of RcCcdA [48]. However, *R. capsulatus* is different from *H. pylori* and *B. subtillius,* in that it has a CcdA and an ScsB. In the publication that describes the *E. coli* DsbD that reduces *R. capsulatus* CcmG in the absence of its CcdA, the authors failed to knock out the ScsB [67]. Due to the structural resemblance, it is possible that ScsB complements the absence of CcdA. Hence, the conclusion that *E. coli* DsbD transfers electrons to the *R. capsulatus* CcmG might have been misleading. The two conserved cysteine residues of CcdA are membrane-embedded, and CcdA lacks the corresponding alpha and gamma domains of DsbD. The mechanism of transmembrane electron transfer by CcdA is still inexplicable. It is likely that it adopts an hourglass-like structure, similar to the Dsb β domain, that allows it to interact with cytoplasmic thioredoxin and its periplasmic partners [68, 69]. We suggest that any DsbD can only transfer electrons in a proteome that has either a DsbD or a ScsB, but not in a proteome that has only a CcdA. The effective transmembrane electron transfer is also determined by the structure of the proteins that are substrates of the envelope DsbD or DsbD–like proteins. The best example is the *Legionella pneumophila* Dsb system. This bacterium produces two DsbA-like proteins (monomeric LpDsbA1 and dimeric LpDsbA2), two DsbBs and two DsbDs. The DsbD-like proteins differ in their structure: LpDsbD1 resembles EcDsbD, whereas LpDsbD2 lacks the α domain present in EcDsbD. LpDsbA2, the bifunctional protein acting as an oxidase and isomerase, interacts with both LpDsbDs and also with EcDsbD, whereas EcDsbC is not reduced by LpDsbD1 or by LpDsbD2 [70, 71]. Therefore, *E. coli* DsbD reduces *Bordetella* CcmG, and not HP0377, because the *Bordetella* genome encodes a DsbD, while the *H. pylori* proteome has a CcdA but no DsbD/ScsB [67]. Similarly, *B. subtilis* has a CcdA but no DsbD/ScsB, and therefore *E. coli* DsbD will not reduce its CcmG.

## Conclusions

The fact that *H. pylori* contains only two classical Dsb proteins, and at the same time, its genome encodes many proteins containing consecutive and nonconsecutive disulfide bonds, implies the presence of an atypical Dsb protein network. The experimental results presented strongly suggest that HP0377, despite a structural similarity to other CcmGs, functions differently from them. Biochemical analysis leads us to conclude that HP0377 is a multifunctional protein. As *H. pylori* possesses several proteins containing nonconsecutive disulfide bonds, we postulate that the activity of HP0377 complements the lack of a classical DsbC. Also, analysis of the HP0377 redox state in wt or *dsb* mutant *H. pylori* cells supports this assumption, as it indicates cooperation between the dimeric oxidoreductase HP0231 and HP0377. However, further biochemical or structural experiments are required to confirm the nature of potential interaction between HP0377 and HP0231, and to identify substrates of HP0377, if any.

## Additional files

Additional file 1: Figure S1. Analysis of redox state of HP0377. Analysis of redox state of 6xHis-HP0377 in the buffer containing 0.1 mM GSSG (oxidized glutathione) and an increasing concentration of GSH (reduced glutathione). Fractions were resolved by (12 %) SDS PAGE, followed by gel staining with Coomassie Brilliant Blue. The diagram represents determination of the redox potential by equilibration against glutathione. The fraction (R) of reduced HP0377 at equilibrium was measured by analysis of redox state, and the intensity of the bands corresponding to the reduced protein compared to intensity corresponding to a protein in both forms was measured by ImageJ program. Additional file 2: Figure S2. The insulin reduction assay. The reaction contained 150 μM insulin in potassium phosphate buffer, pH 7.0 and 2mM EDTA. The reaction was performed in the absence or presence of 10 μM EcDsbA, 10 μM HP0377. Reactions started by adding DTT to the final concentration of 1 mM. The changes in the absorbance at 650 nm as a function of time were measured. Three independent experiments were performed. Additional file 3: Figure S3. Oxidase activity assay. The reaction contained 10 μM reduced RNase in 100 mM Tris acetate, pH 8.0, 2 mM EDTA, 0.2 mM GSSG, 1 mM GSH, and 9 mM cCMP. The reaction was performed in the absence or presence of 20 μM EcDsbA or 20 μM HP0377. The changes in absorbance at 296 nm were measured as a function of time. Three independent experiments were performed. Additional file 4: Figure S4. Production of HP0377 in *E. coli*. Plasmid pUWM399 was introduced to *E. coli*. The expression of the heterologous gene was confirmed by Western blot using anti-HP0377 antibody. Lanes: 1 – protein ladder, 2 – *E. coli dsbA*::*kan*, 3 – *E. coli dsbA*::*kan1* with empty pHEL2, 4 - *E. coli dsbA*::*kan1* with pUWM399 (*) unspecific signals recognized by anti-HP0377 antibody. Additional file 5: Figure S5. HP0377 doesn’t restore the *E. coli dsbA* ^−^ wild type phenotype in three independent functional assays. As a negative control *E. coli dsbA*::*aph* was transformed with an empty pHEL2 vector. (A) motility assay; (B) alkaline phosphatase assay. (C) DTT sensitivity assay. Additional file 6: Figure S6. HP0377 does not restore the *E. coli dsbC* ^**−**^ wild type phenotype in the copper sensitive assays. As a negative control *E. coli dsbC*::*aph* was transformed with an empty pHEL2 vector. The numbers indicate: 1 – WT, 2 - *dsbC*::*kan*, 3 *– dsbC*::*kan/hp0231* ^*+*^, 4 *– dsbC*::*kan/hp0377*, 5 *– dsbC*::*kan/*pHEL2. Additional file 7: Figure S7. Production of HP0265 is lethal in *E. coli.* The diagrams show growth curves of different strains of *E. coli* : PR580(*dsbD* ^*−*^_,_ *hp0377* ^*+*^), PR581 (wt, *hp0377* ^+^), PR539(*dsbD* ^*−*^_*,*_ *hp0377* ^*+*^ *, hp0265* under arabinose promotor), PR540 (wt_,_ *hp0377* ^*+*^ *, hp0265* under arabinose promotor. Bacteria were grown in LB at 37 °C without (A) or with addition of 0.2 % arabinose (B) at the time indicated with a black arrow.
