# Supplementary figures and images for: Helicobacter pylori HP0377, a member of the Dsb family, is an untypical multifunctional CcmG that cooperates with dimeric thioldisulfide oxidase HP0231
Source: BMC Microbiol. 2015 Jul 4;15:135. doi: 10.1186/s12866-015-0471-z (PMC4491210; doi:10.1186/s12866-015-0471-z)

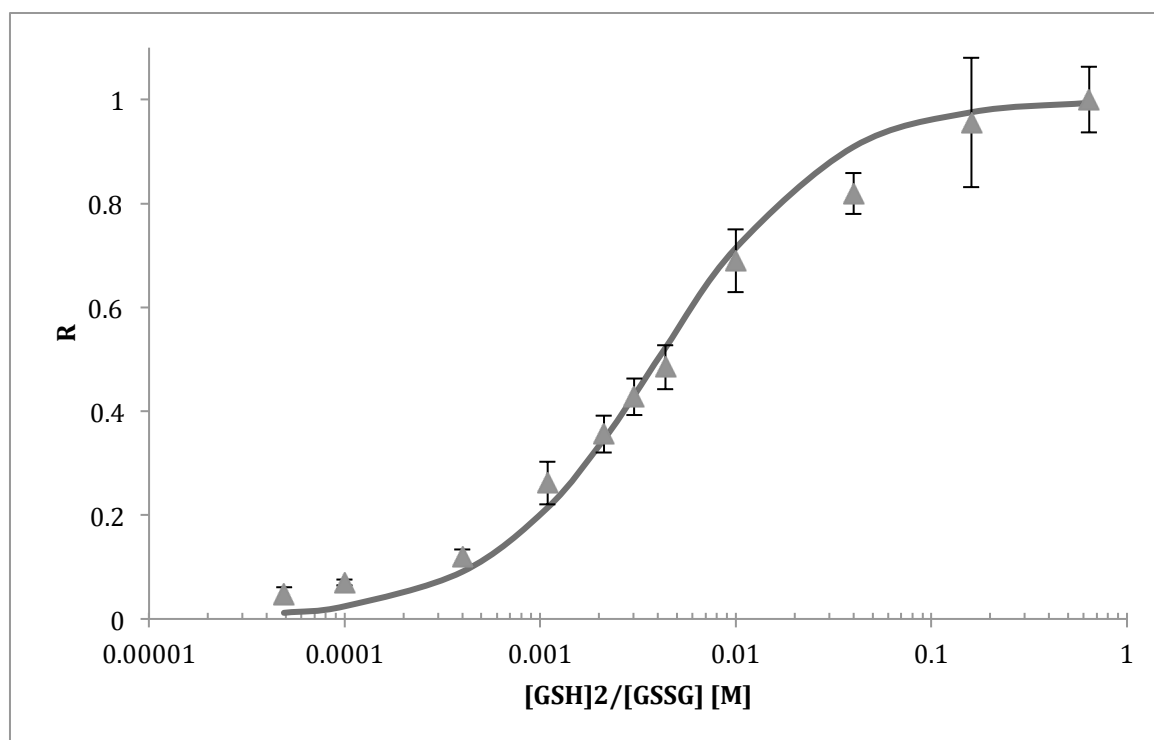

Supplement: Additional file 1: Figure S1. — Analysis of redox state of HP0377. Analysis of redox state of 6xHis-HP0377 in the buffer containing 0.1 mM GSSG (oxidized glutathione) and an increasing concentration of GSH (reduced glutathione). Fractions were resolved by (12 %) SDS PAGE, followed by gel staining with Coomassie Brilliant Blue. The diagram represents determination of the redox potential by equilibration against glutathione. The fraction (R) of reduced HP0377 at equilibrium was measured by analysis of redox state, and the intensity of the bands corresponding to the reduced protein compared to intensity corresponding to a protein in both forms was measured by ImageJ program. [file 12866_2015_471_MOESM1_ESM.pdf]

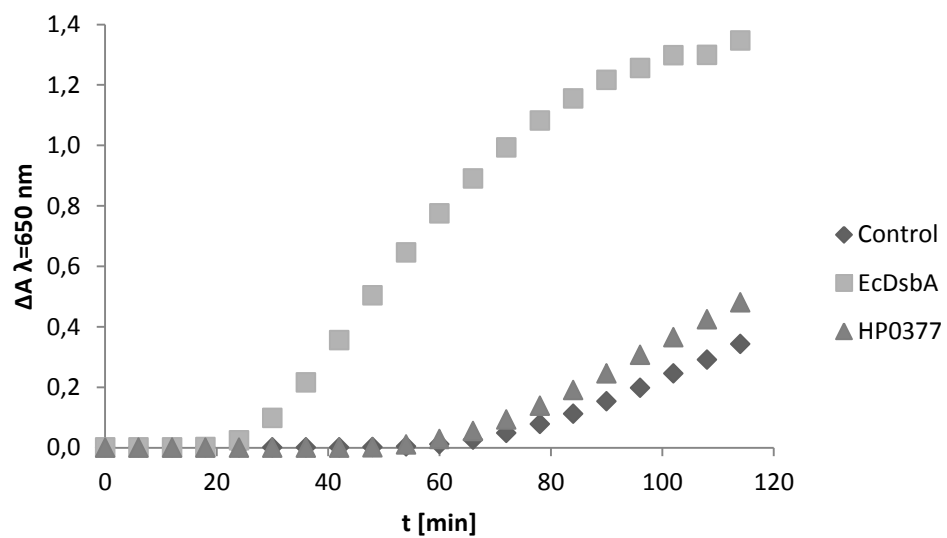

Supplement: Additional file 2: Figure S2. — The insulin reduction assay. The reaction contained 150 μM insulin in potassium phosphate buffer, pH 7.0 and 2mM EDTA. The reaction was performed in the absence or presence of 10 μM EcDsbA, 10 μM HP0377. Reactions started by adding DTT to the final concentration of 1 mM. The changes in the absorbance at 650 nm as a function of time were measured. Three independent experiments were performed. [file 12866_2015_471_MOESM2_ESM.pdf]

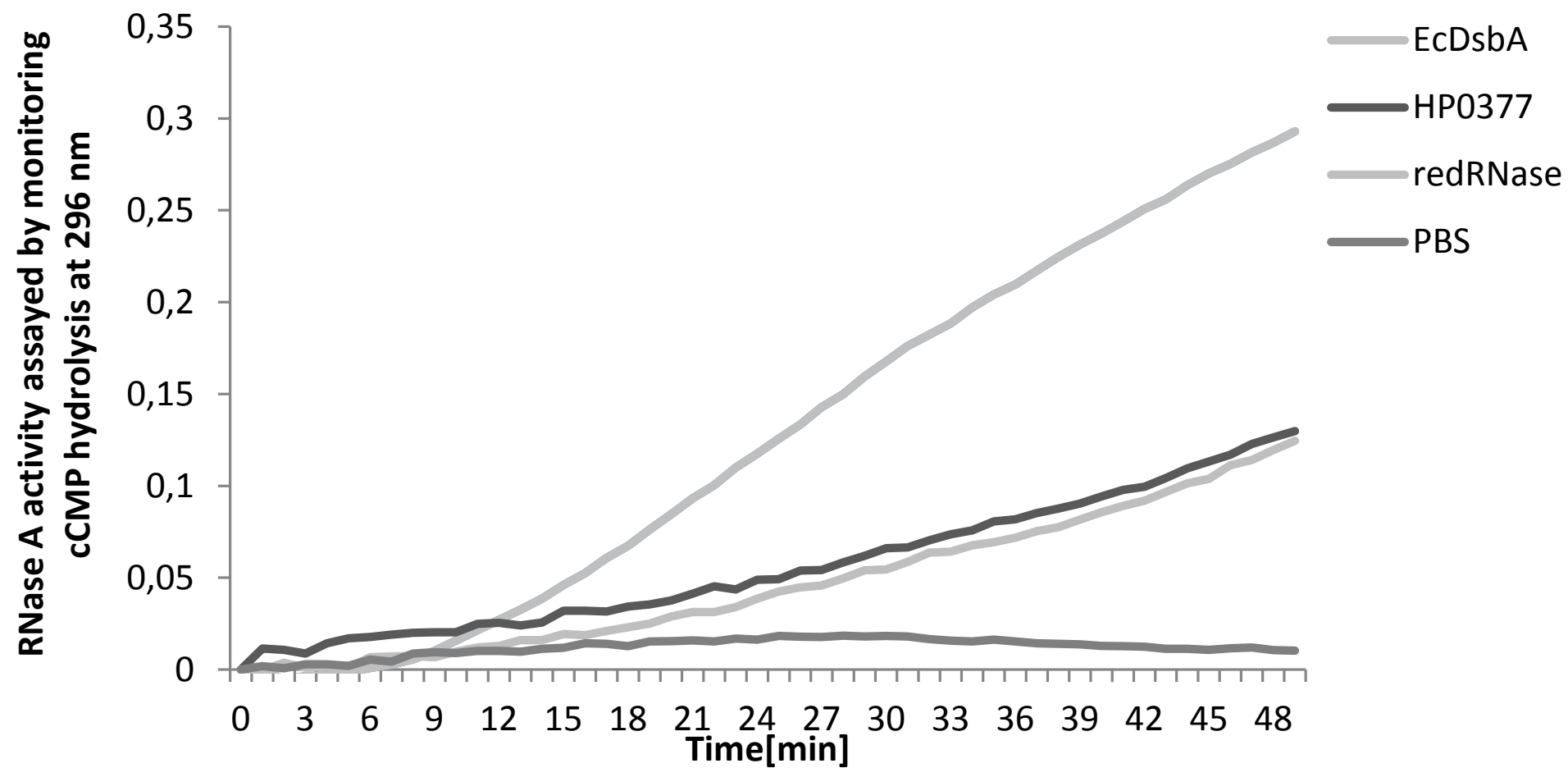

Supplement: Additional file 3: Figure S3. — Oxidase activity assay. The reaction contained 10 μM reduced RNase in 100 mM Tris acetate, pH 8.0, 2 mM EDTA, 0.2 mM GSSG, 1 mM GSH, and 9 mM cCMP. The reaction was performed in the absence or presence of 20 μM EcDsbA or 20 μM HP0377. The changes in absorbance at 296 nm were measured as a function of time. Three independent experiments were performed. [file 12866_2015_471_MOESM3_ESM.pdf]

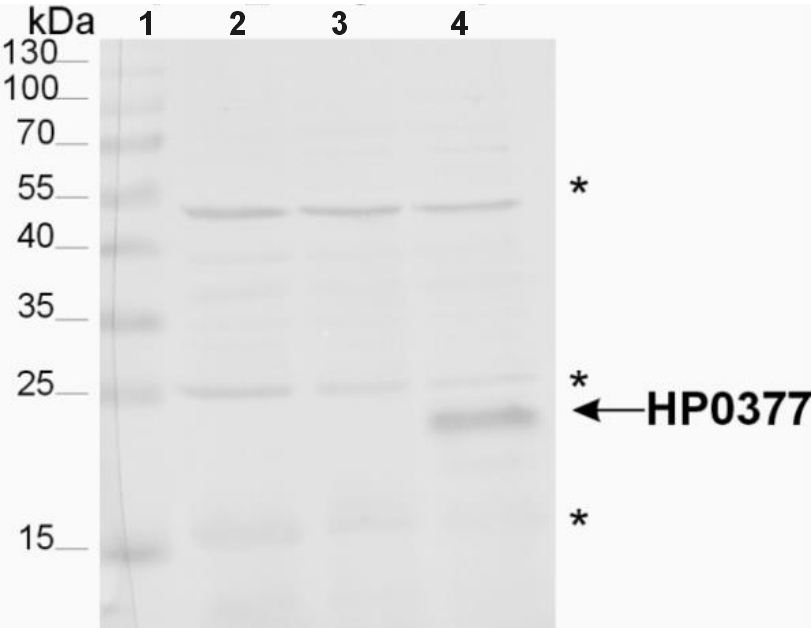

Supplement: Additional file 4: Figure S4. — Production of HP0377 in E. coli. Plasmid pUWM399 was introduced to E. coli. The expression of the heterologous gene was confirmed by Western blot using anti-HP0377 antibody. Lanes: 1 – protein ladder, 2 – E. coli dsbA::kan, 3 – E. coli dsbA::kan1 with empty pHEL2, 4 - E. coli dsbA::kan1 with pUWM399 (*) unspecific signals recognized by anti-HP0377 antibody. [file 12866_2015_471_MOESM4_ESM.tiff]

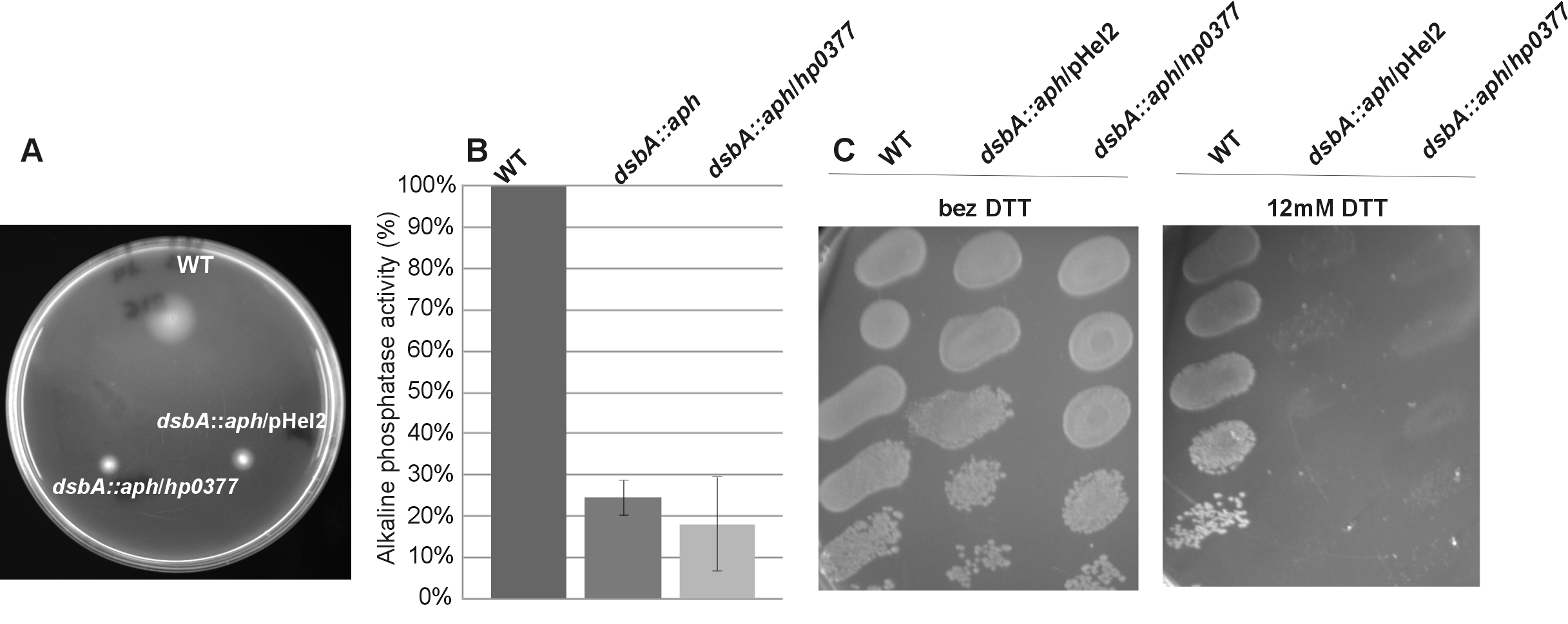

Supplement: Additional file 5: Figure S5. — HP0377 doesn’t restore the E. coli dsbA − wild type phenotype in three independent functional assays. As a negative control E. coli dsbA::aph was transformed with an empty pHEL2 vector. (A) motility assay; (B) alkaline phosphatase assay. (C) DTT sensitivity assay. [file 12866_2015_471_MOESM5_ESM.tiff]

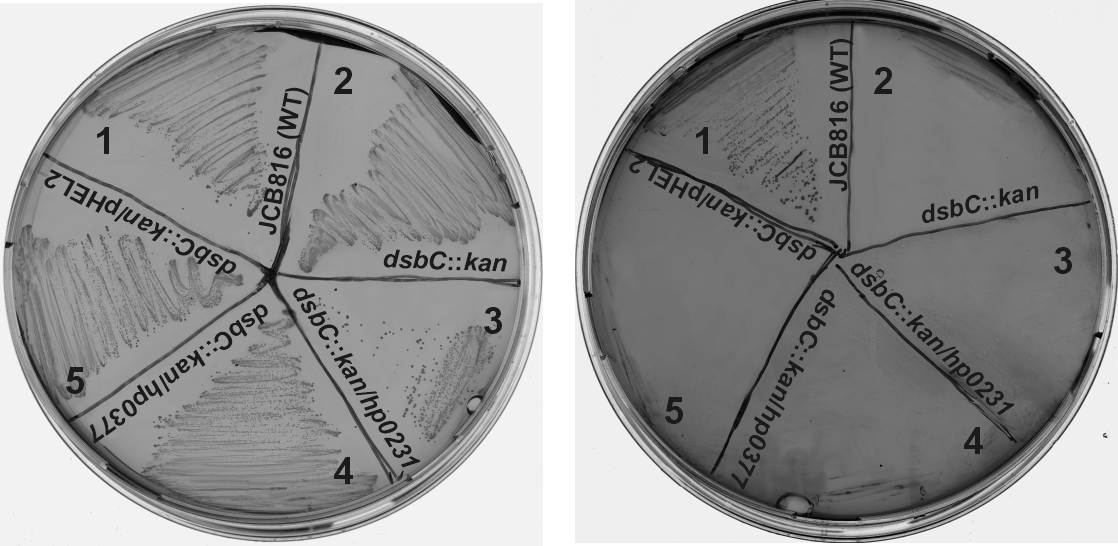

Supplement: Additional file 6: Figure S6. — HP0377 does not restore the E. coli dsbC − wild type phenotype in the copper sensitive assays. As a negative control E. coli dsbC::aph was transformed with an empty pHEL2 vector. The numbers indicate: 1 – WT, 2 - dsbC::kan, 3 – dsbC::kan/hp0231 +, 4 – dsbC::kan/hp0377, 5 – dsbC::kan/pHEL2. [file 12866_2015_471_MOESM6_ESM.tiff]

**A**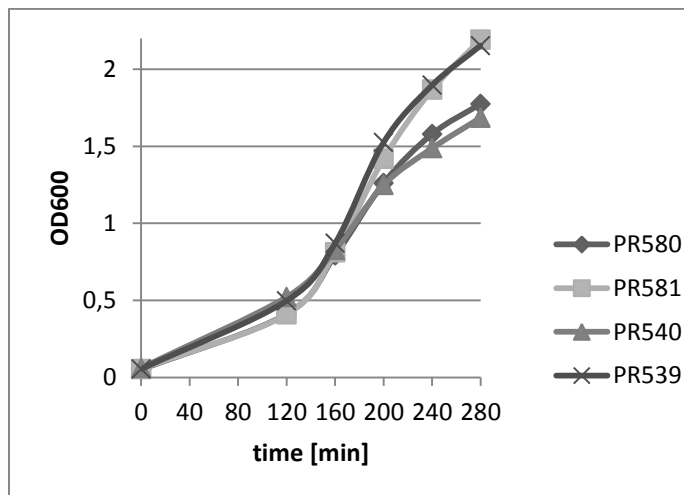**B**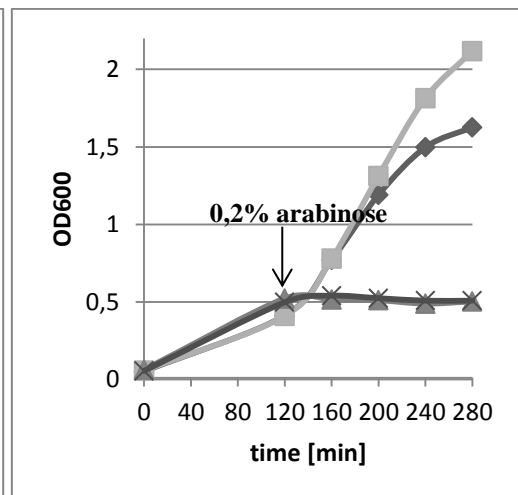

Supplement: Additional file 7: Figure S7. — Production of HP0265 is lethal in E. coli. The diagrams show growth curves of different strains of E. coli : PR580(dsbD − , hp0377 +), PR581 (wt, hp0377 +), PR539(dsbD − , hp0377 + , hp0265 under arabinose promotor), PR540 (wt, hp0377 + , hp0265 under arabinose promotor. Bacteria were grown in LB at 37 °C without (A) or with addition of 0.2 % arabinose (B) at the time indicated with a black arrow. [file 12866_2015_471_MOESM7_ESM.pdf]
